# Supplementary figures and images for: Short-chain fatty acids activate acetyltransferase p300
Source: eLife. 2021 Oct 22;10:e72171. doi: 10.7554/eLife.72171 (PMC8585482; doi:10.7554/eLife.72171)

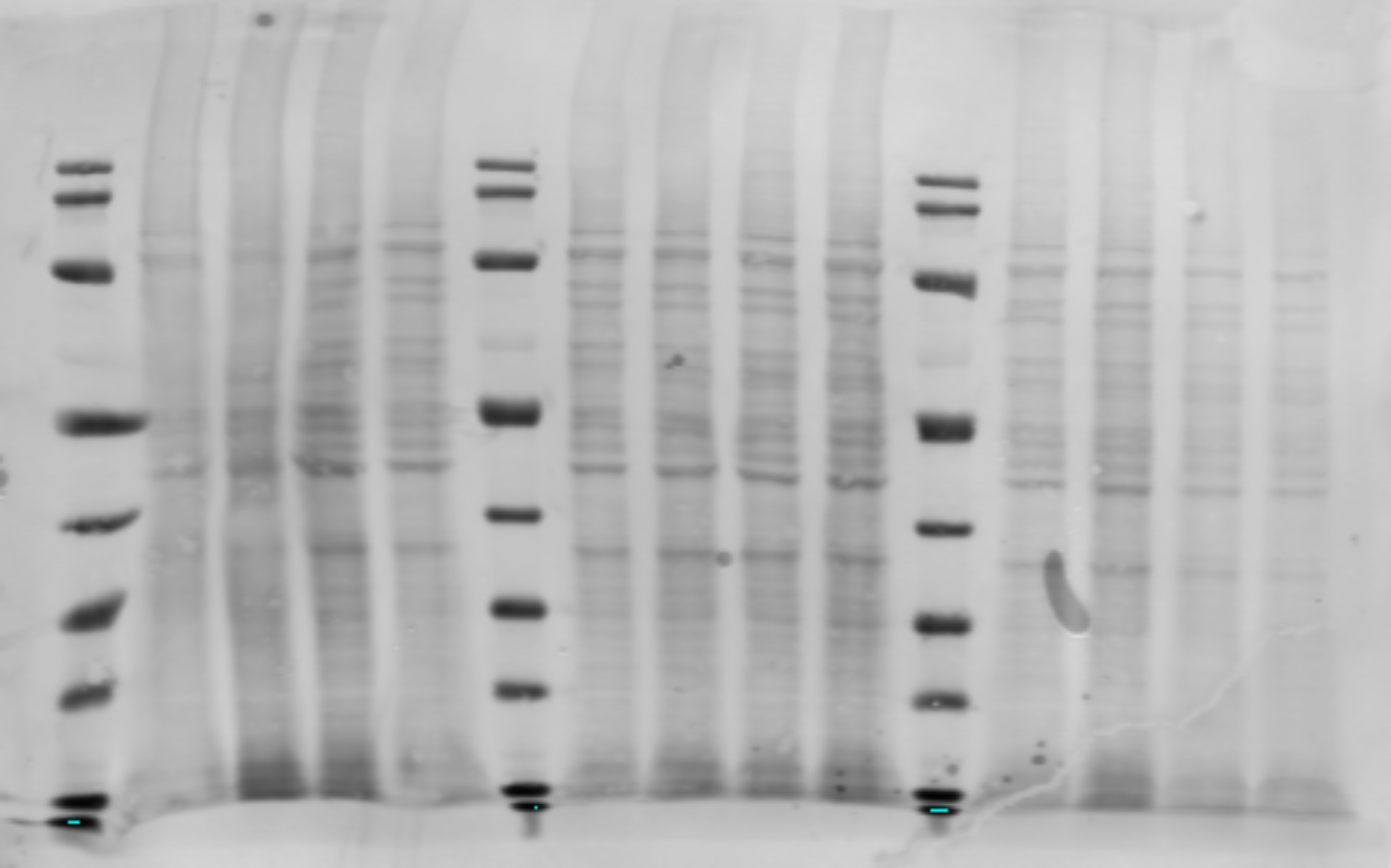

Supplement: Source data 2. [file elife-72171-supp2.zip › SCFA Paper/Supplemental Figure 3C_SourceData1.tif]

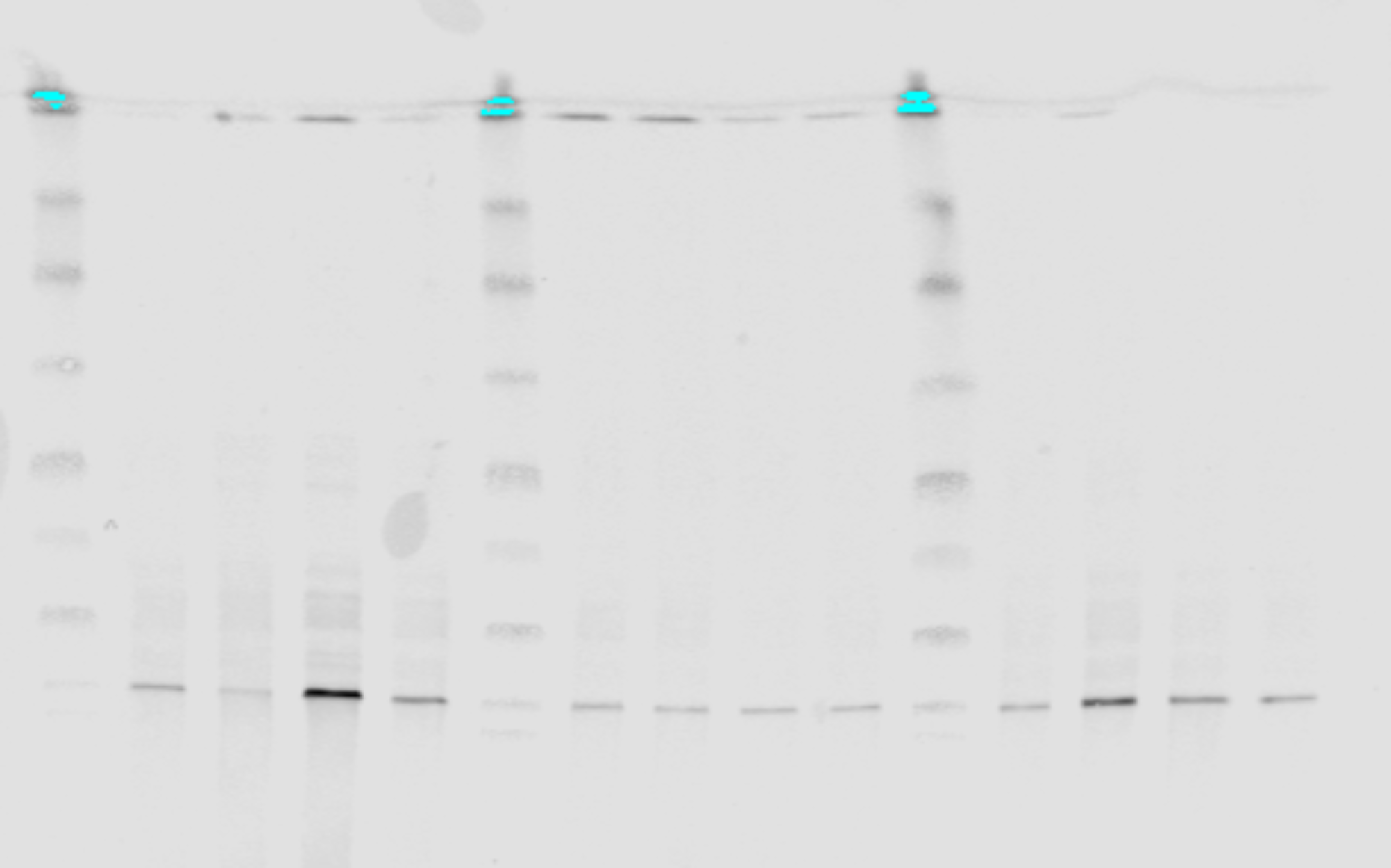

Supplement: Source data 2. [file elife-72171-supp2.zip › SCFA Paper/Supplemental Figure 3C_SourceData2.tif]

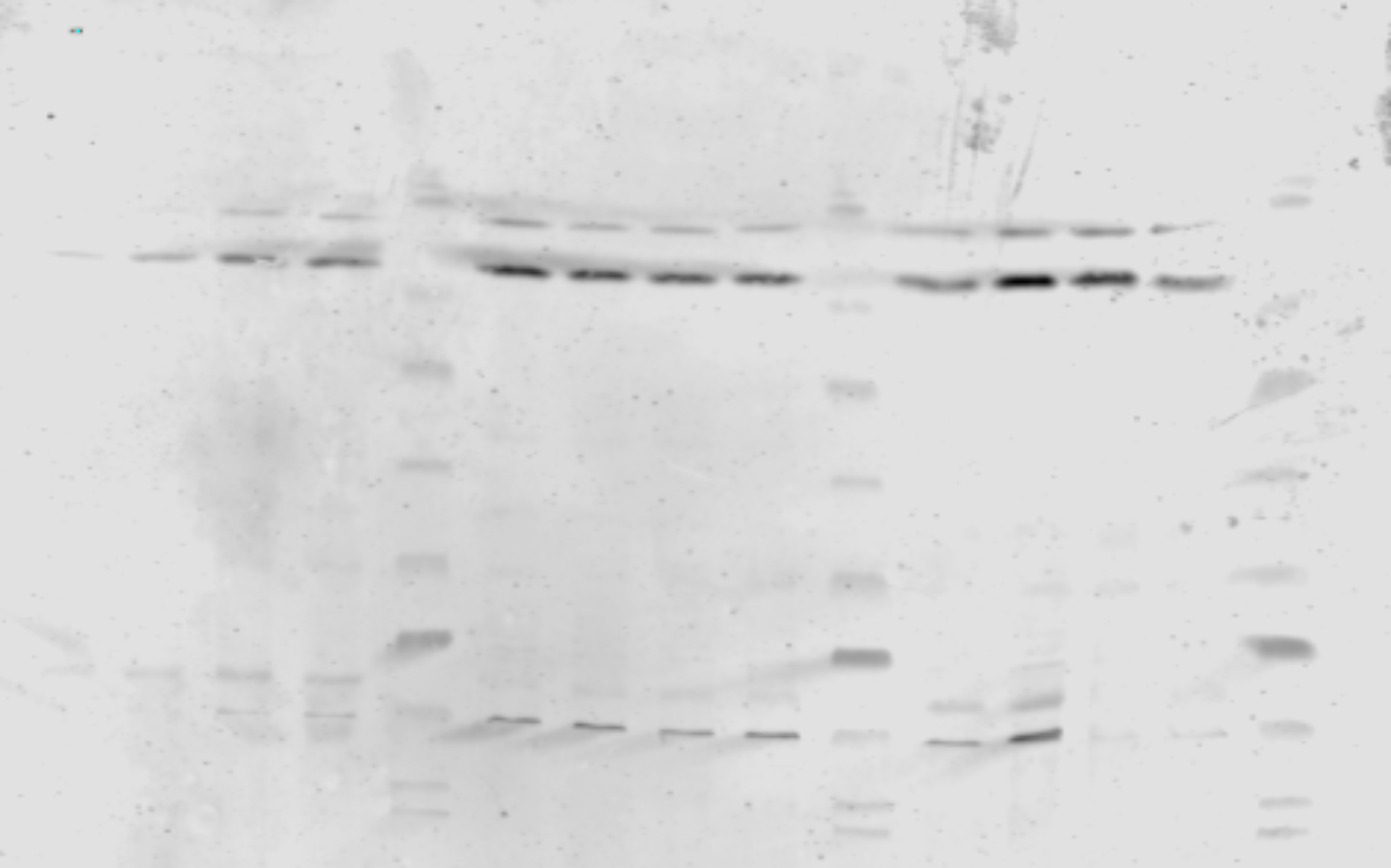

Supplement: Source data 2. [file elife-72171-supp2.zip › SCFA Paper/Supplemental Figure 3C_SourceData3.tif]

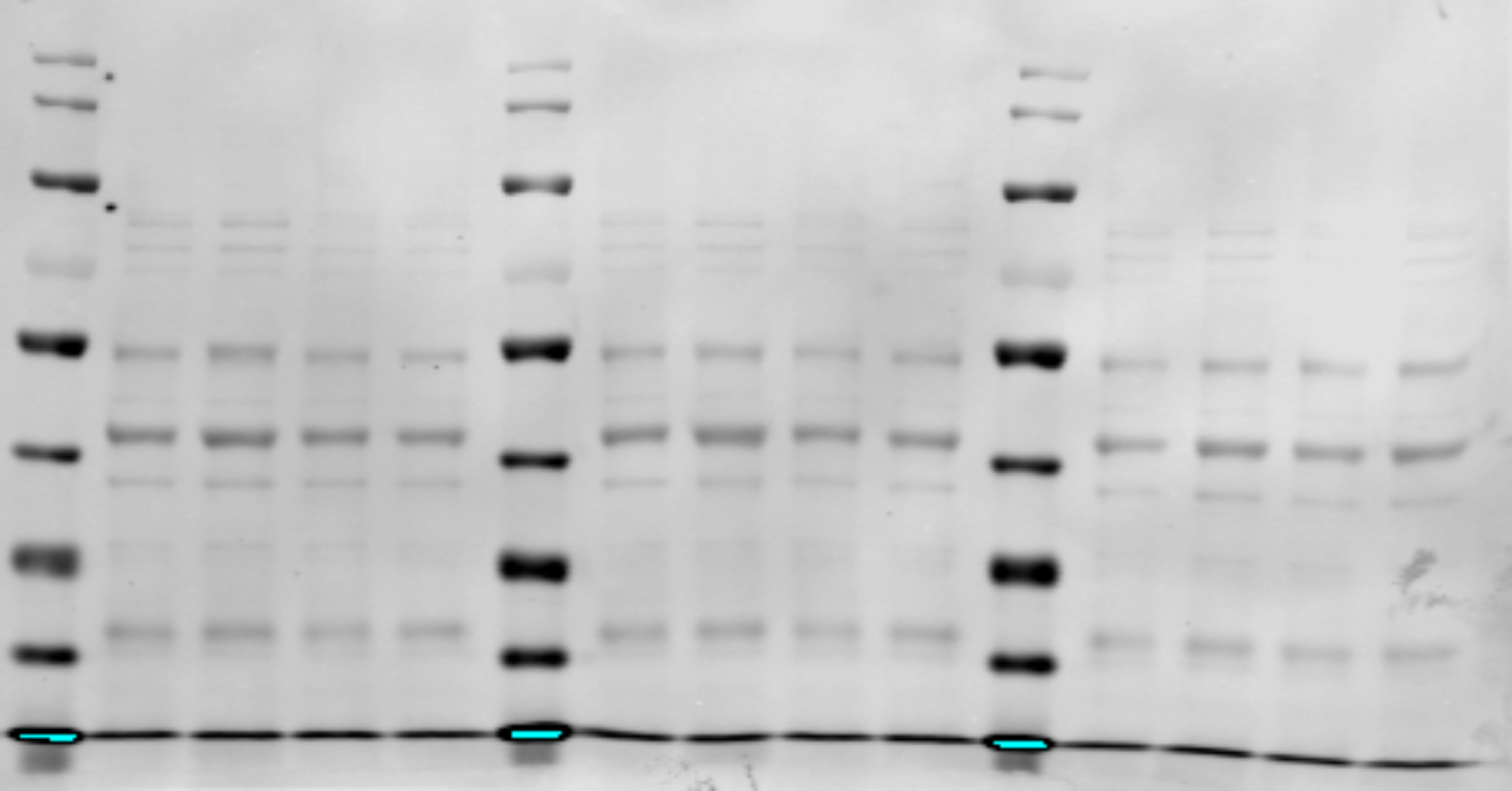

Supplement: Source data 2. [file elife-72171-supp2.zip › SCFA Paper/Supplemental Figure 4A_SourceData1.tif]

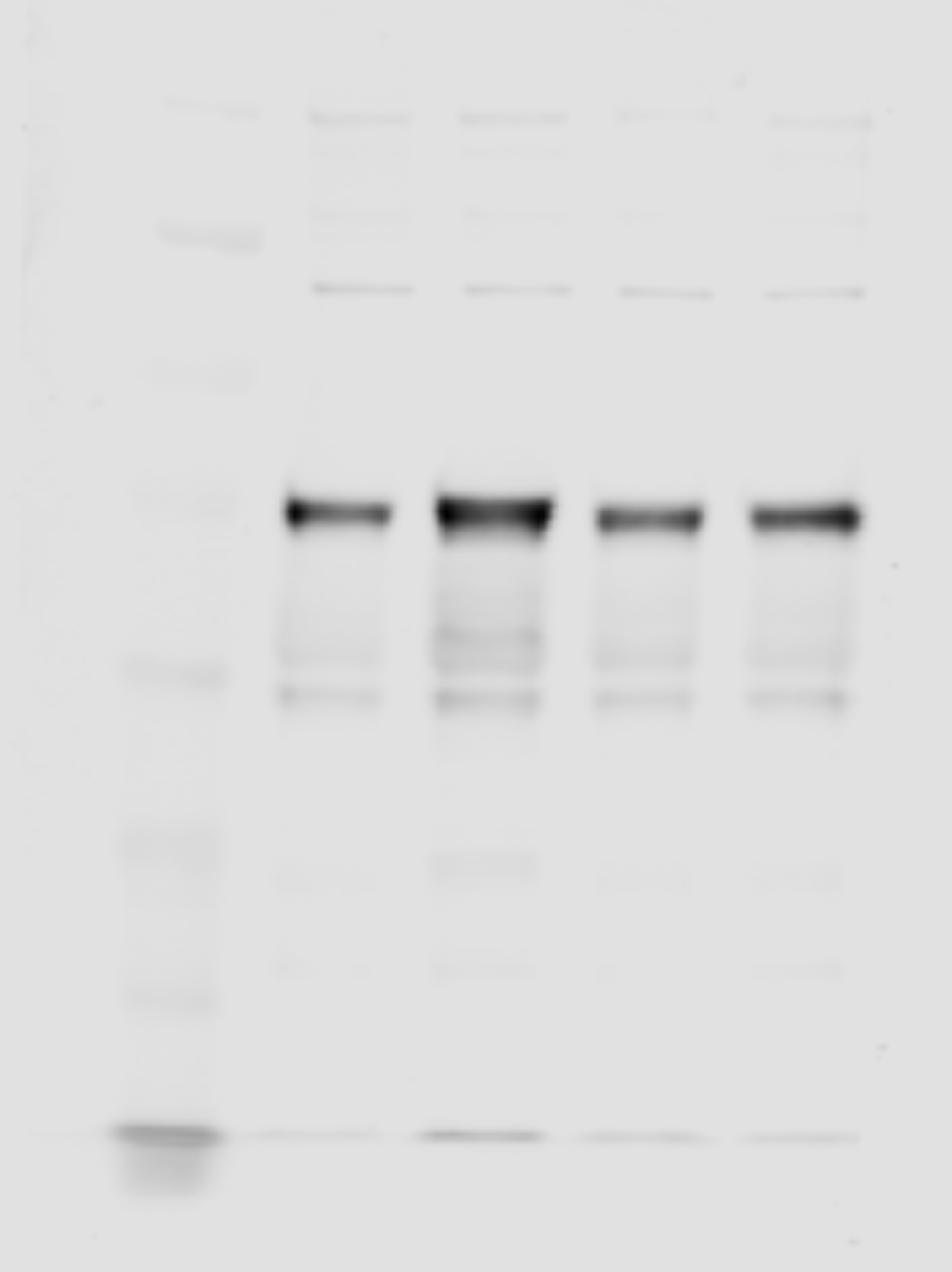

Supplement: Source data 2. [file elife-72171-supp2.zip › SCFA Paper/Supplemental Figure 4A_SourceData2.tif]

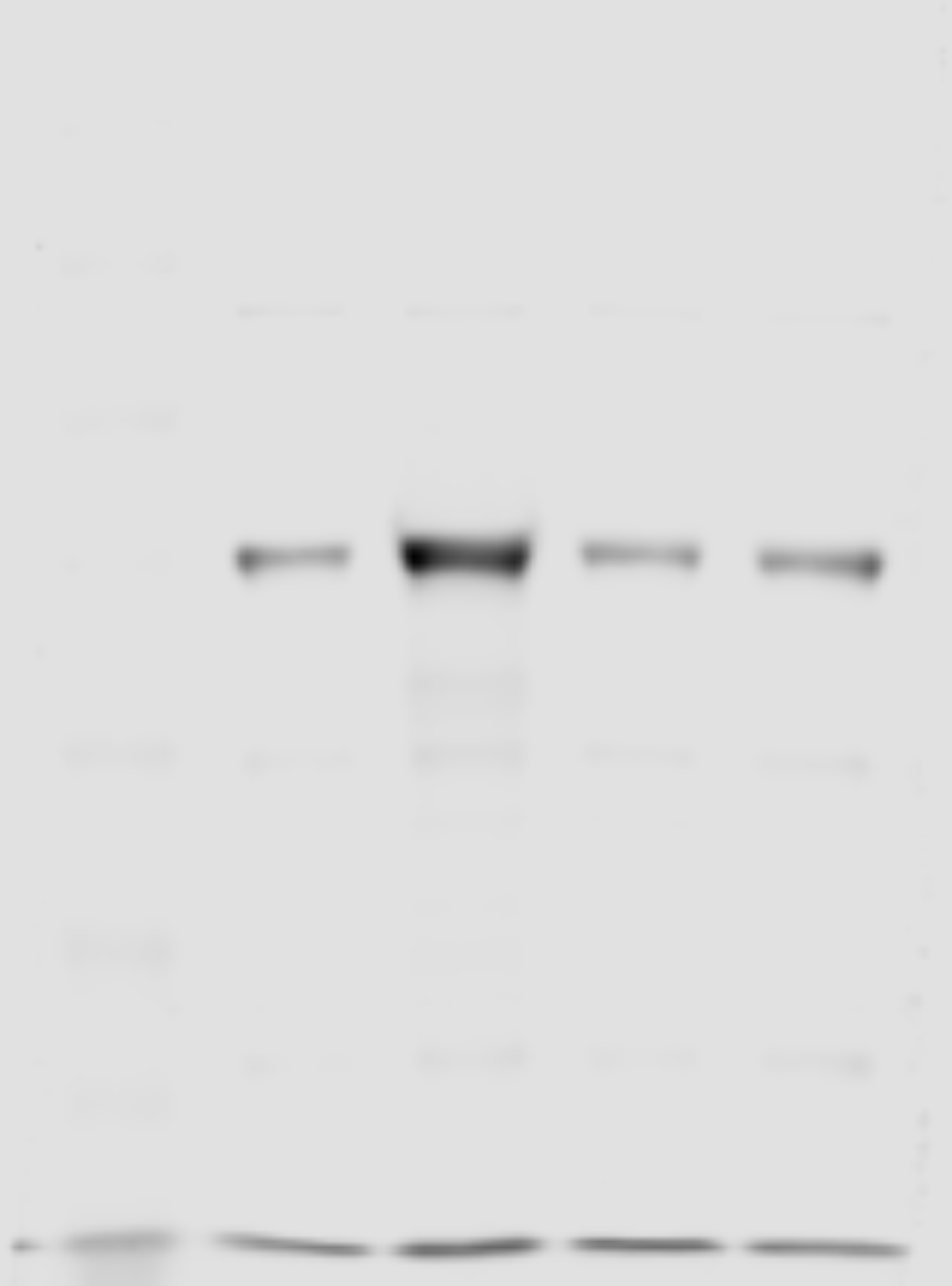

Supplement: Source data 2. [file elife-72171-supp2.zip › SCFA Paper/Supplemental Figure 4A_SourceData3.tif]

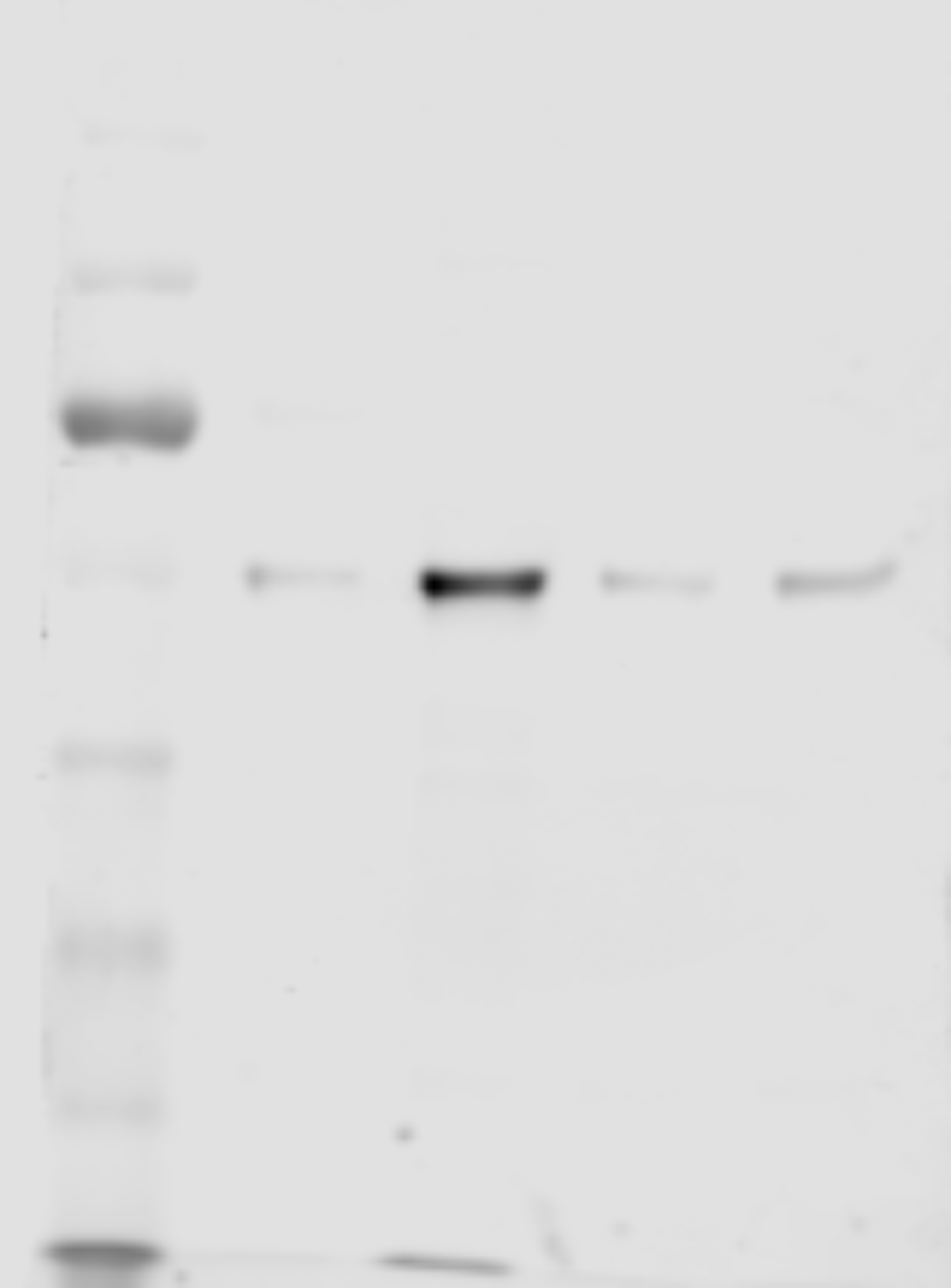

Supplement: Source data 2. [file elife-72171-supp2.zip › SCFA Paper/Supplemental Figure 4A_SourceData4.tif]

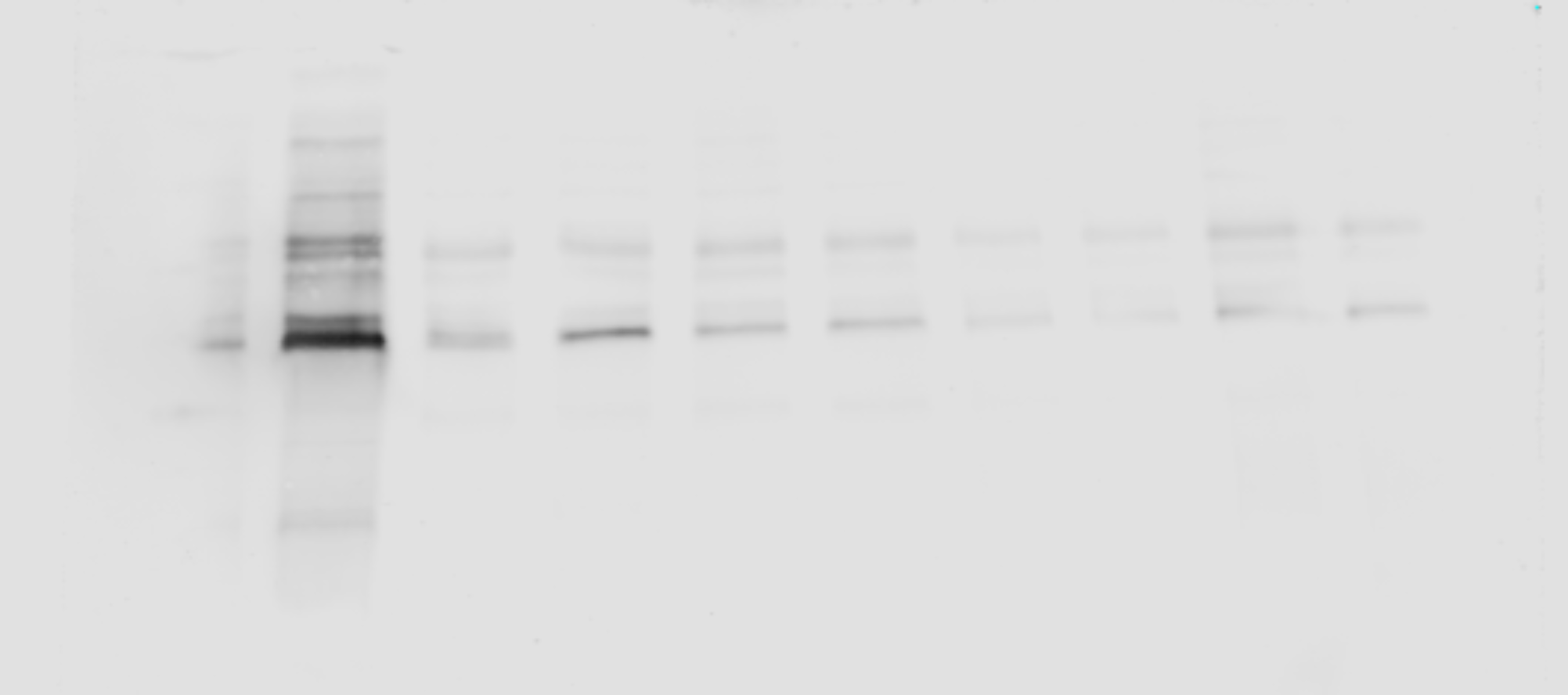

Supplement: Source data 2. [file elife-72171-supp2.zip › SCFA Paper/Supplemental Figure 4B_SourceData1.tif]

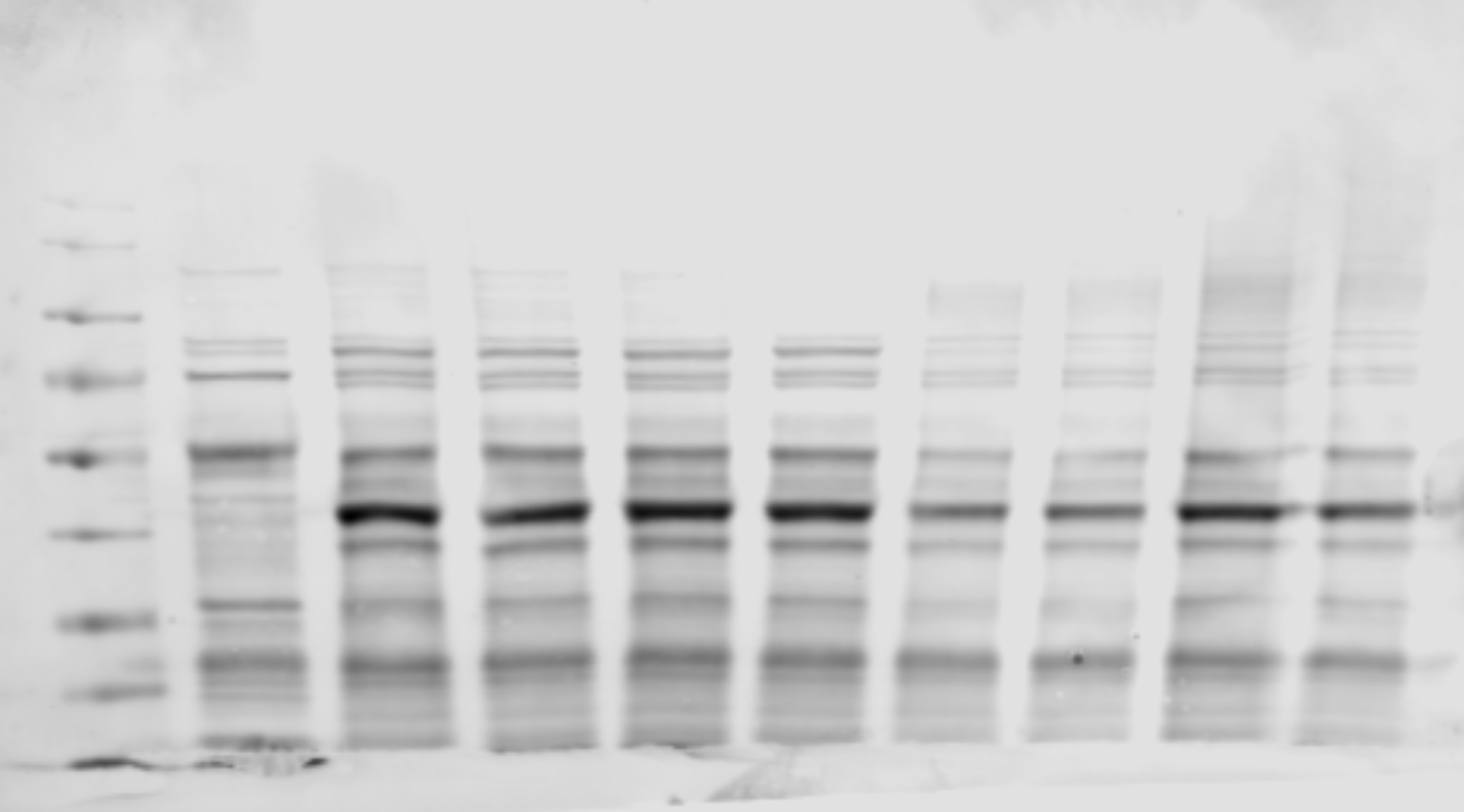

Supplement: Source data 2. [file elife-72171-supp2.zip › SCFA Paper/Supplemental Figure 4B_SourceData2.tif]

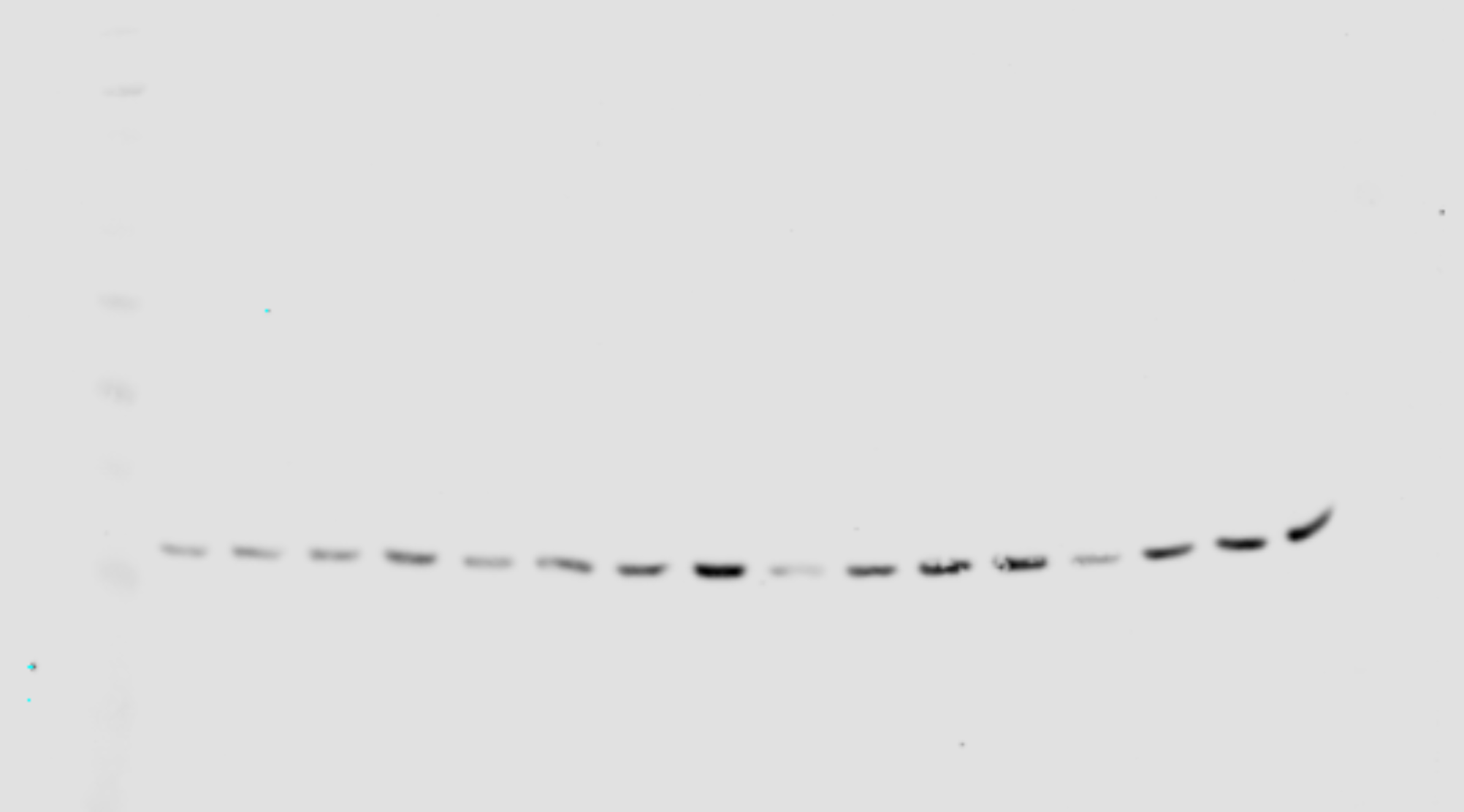

Supplement: Source data 2. [file elife-72171-supp2.zip › SCFA Paper/Supplemental Figure 4C_SourceData1.tif]

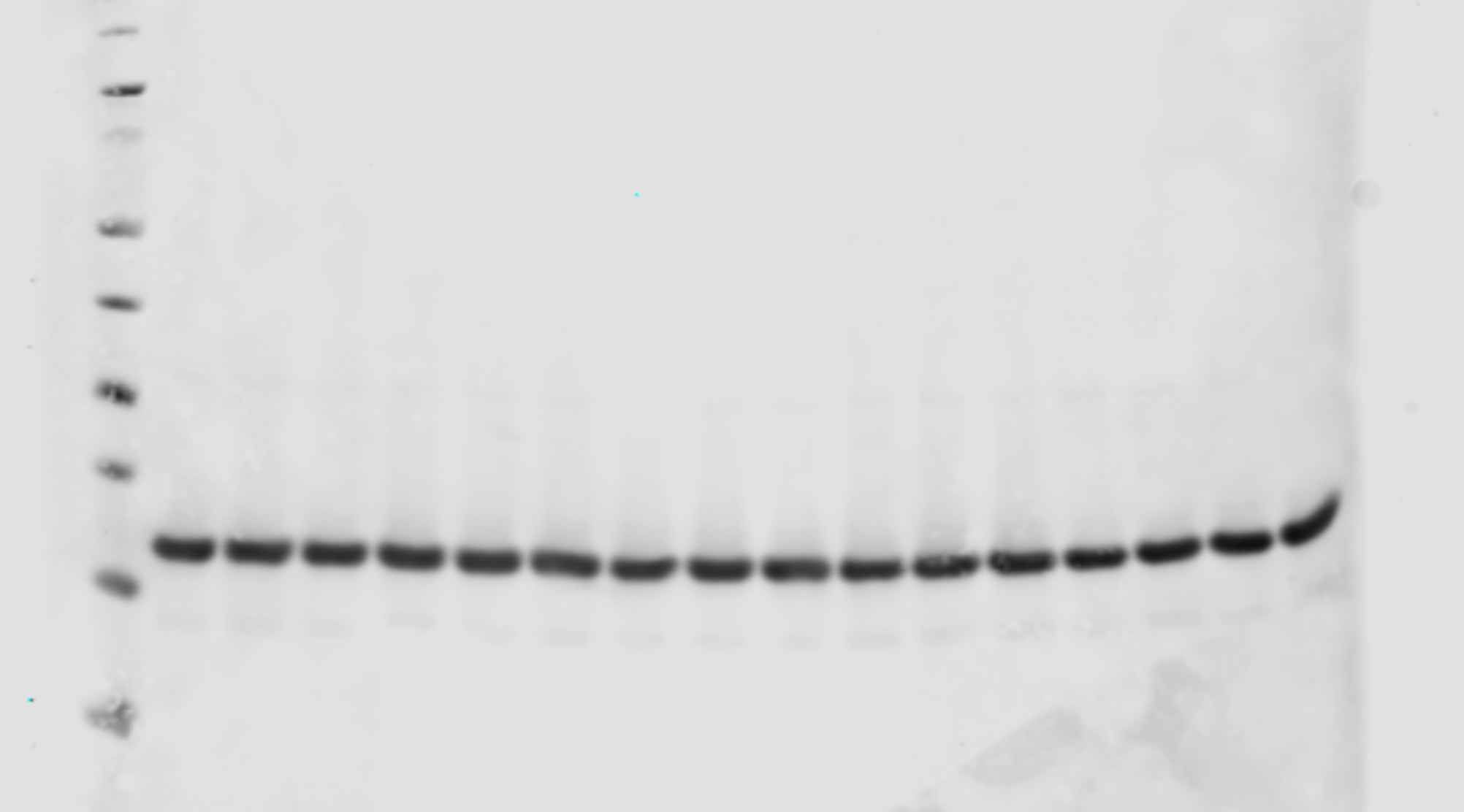

Supplement: Source data 2. [file elife-72171-supp2.zip › SCFA Paper/Supplemental Figure 4C_SourceData2.tif]

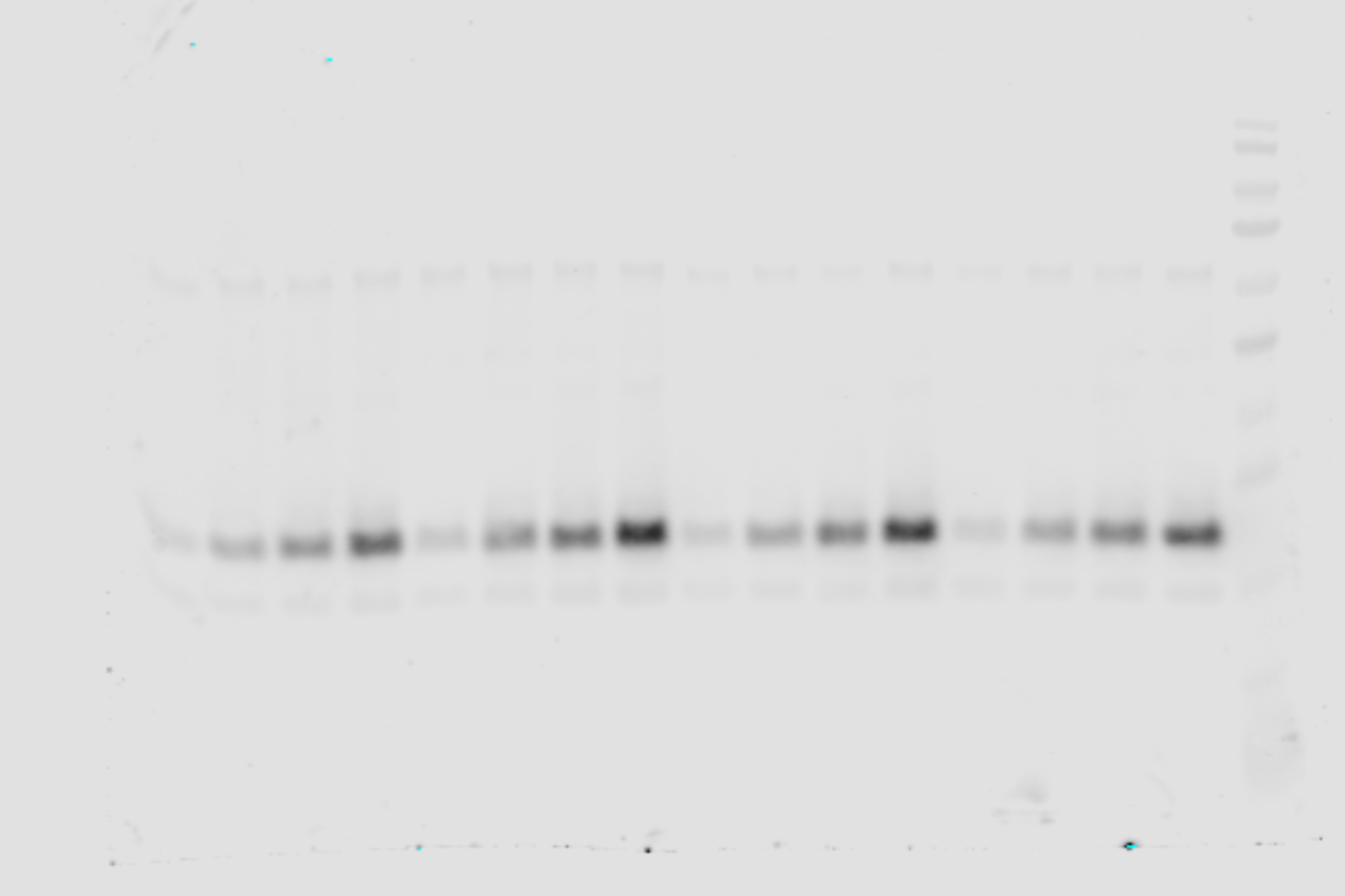

Supplement: Source data 2. [file elife-72171-supp2.zip › SCFA Paper/Supplemental Figure 4C_SourceData3.tif]

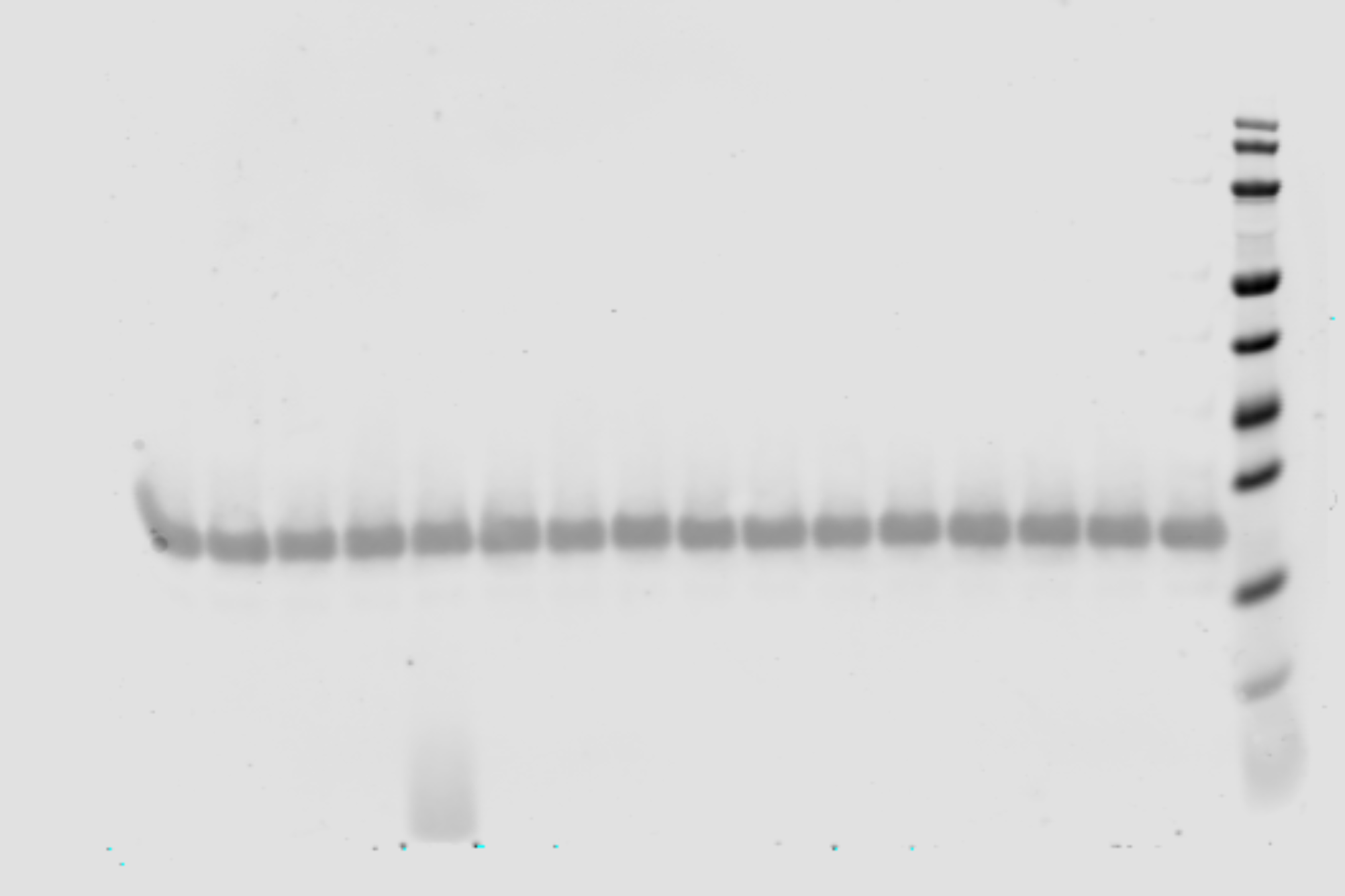

Supplement: Source data 2. [file elife-72171-supp2.zip › SCFA Paper/Supplemental Figure 4C_SourceData4.tif]

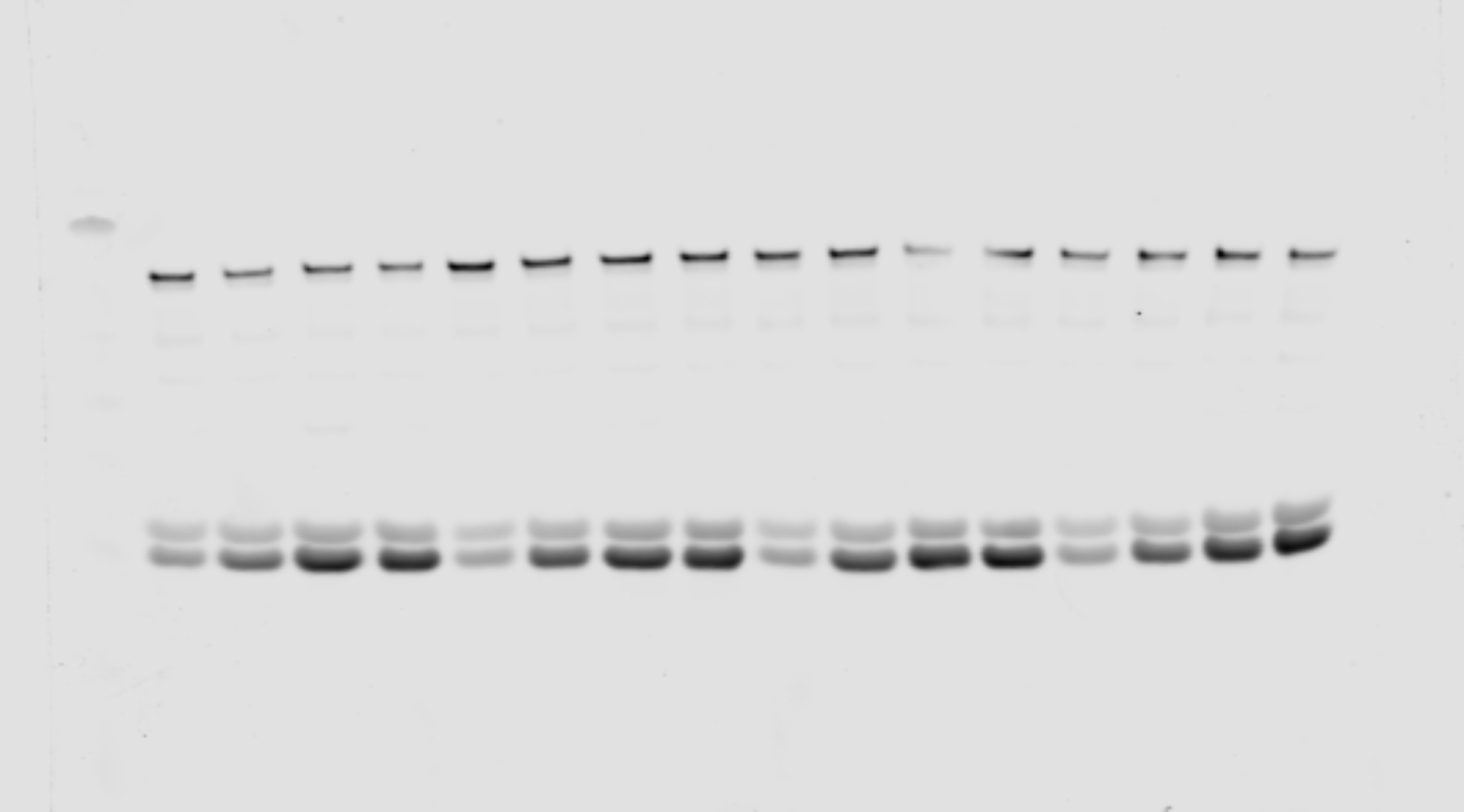

Supplement: Source data 2. [file elife-72171-supp2.zip › SCFA Paper/Supplemental Figure 4C_SourceData5.tif]

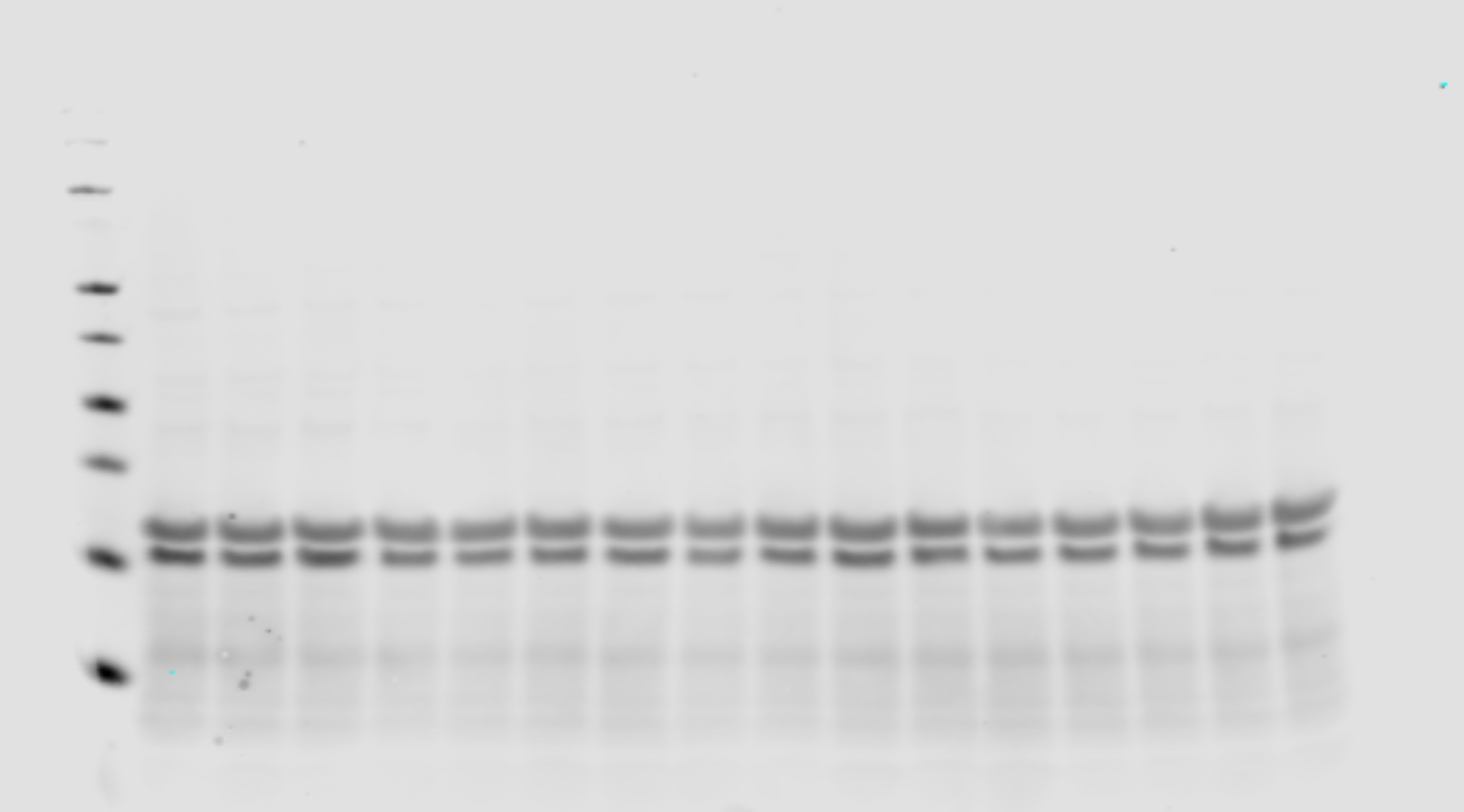

Supplement: Source data 2. [file elife-72171-supp2.zip › SCFA Paper/Supplemental Figure 4C_SourceData6.tif]

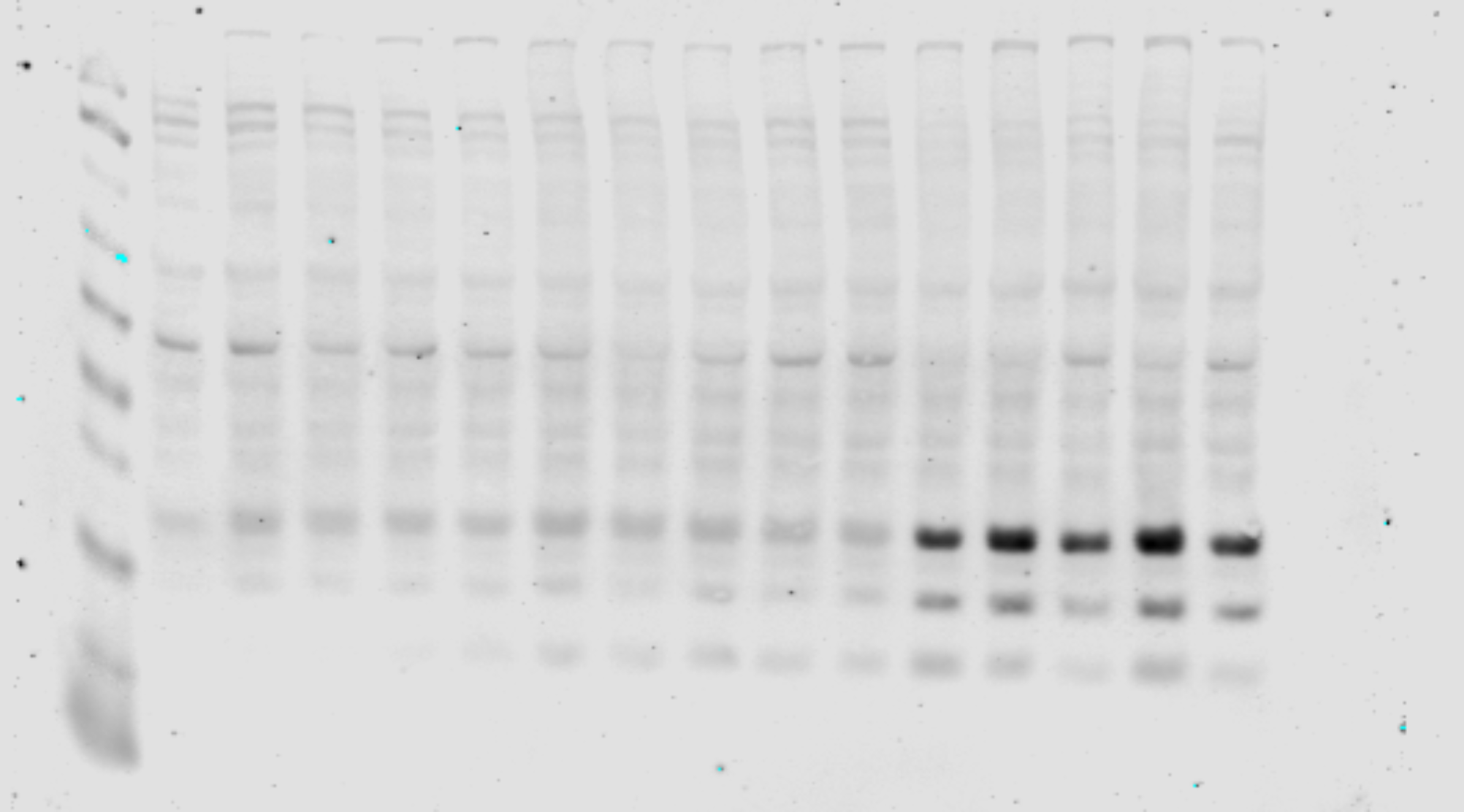

Supplement: Source data 2. [file elife-72171-supp2.zip › SCFA Paper/Supplemental Figure 5A_SourceData1.tif]

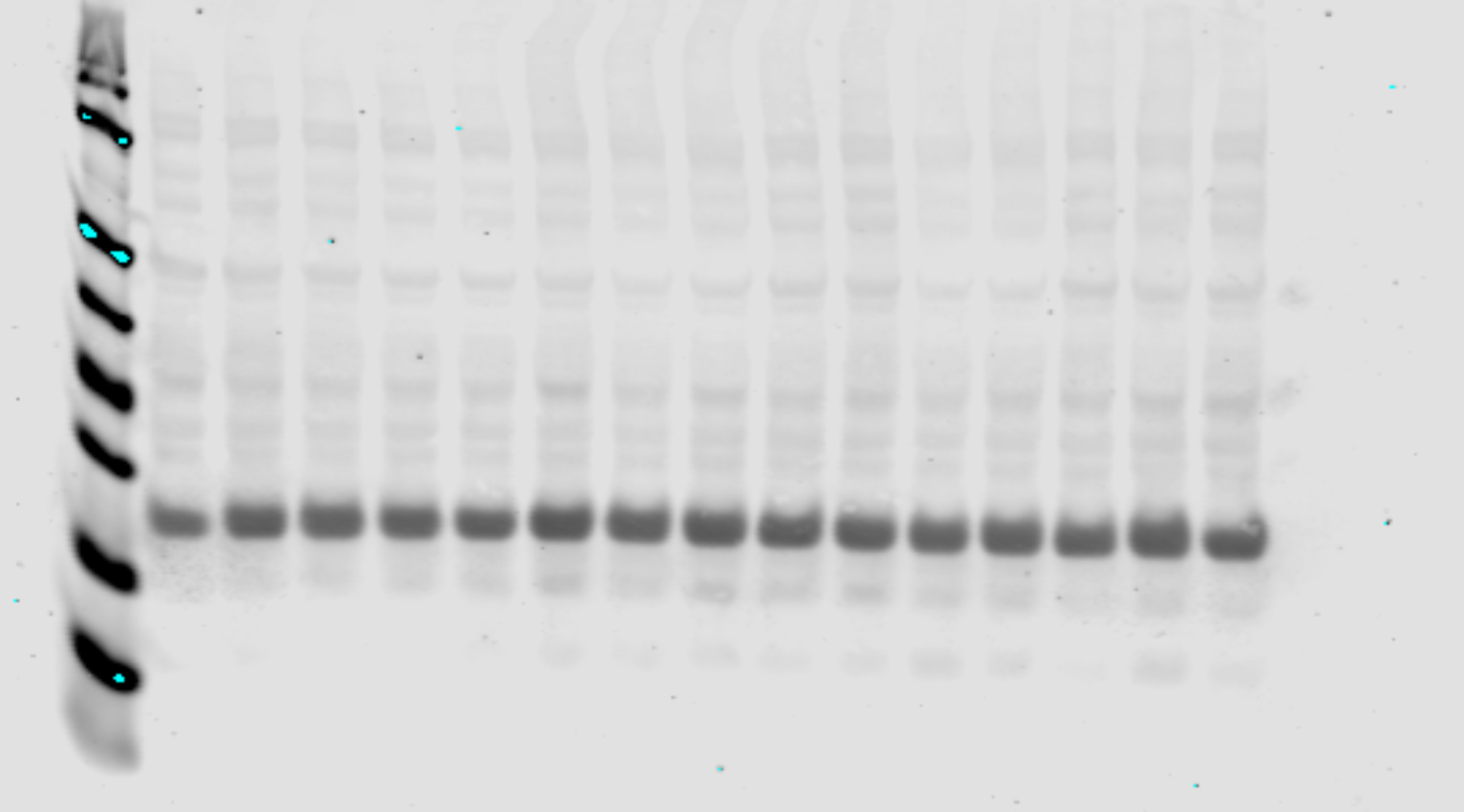

Supplement: Source data 2. [file elife-72171-supp2.zip › SCFA Paper/Supplemental Figure 5A_SourceData2.tif]

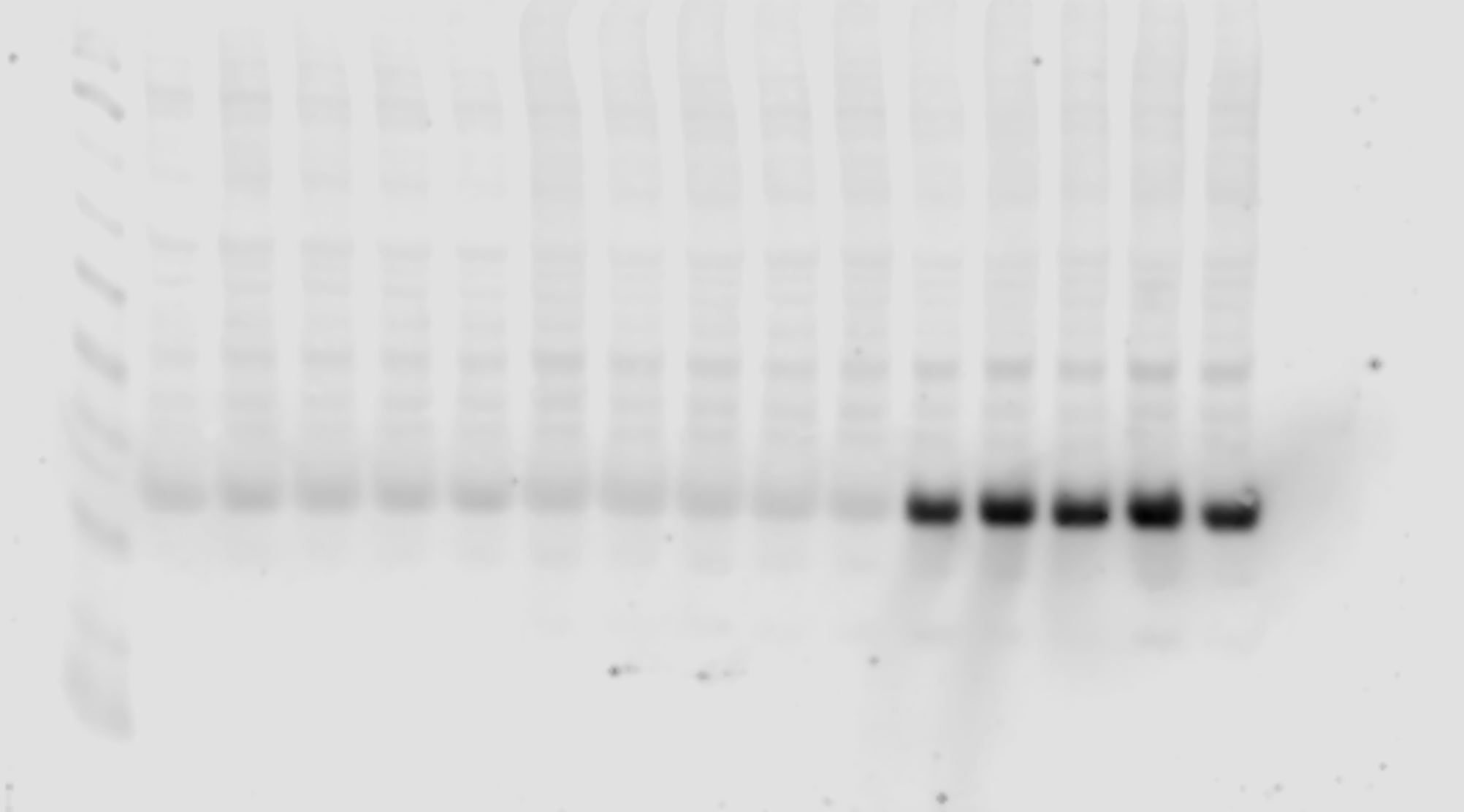

Supplement: Source data 2. [file elife-72171-supp2.zip › SCFA Paper/Supplemental Figure 5A_SourceData3.tif]

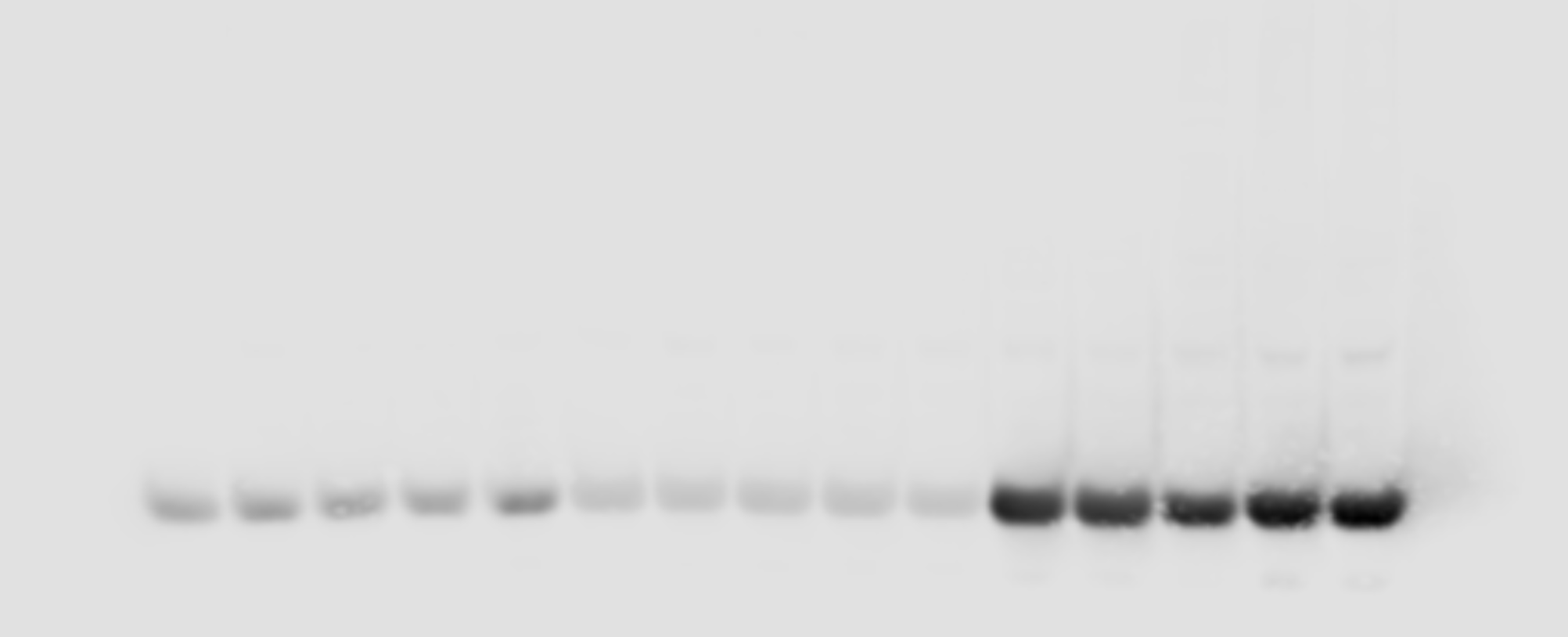

Supplement: Source data 2. [file elife-72171-supp2.zip › SCFA Paper/Supplemental Figure 5A_SourceData4.tif]

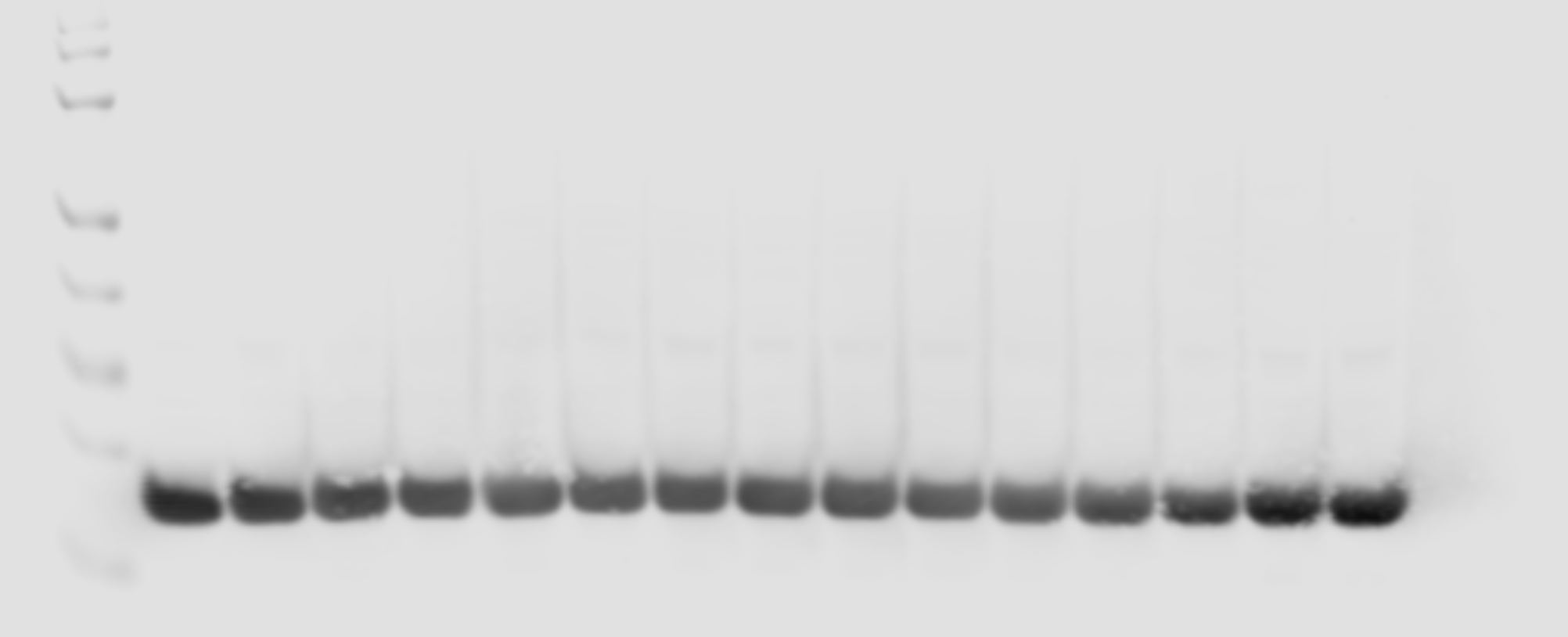

Supplement: Source data 2. [file elife-72171-supp2.zip › SCFA Paper/Supplemental Figure 5A_SourceData5.tif]

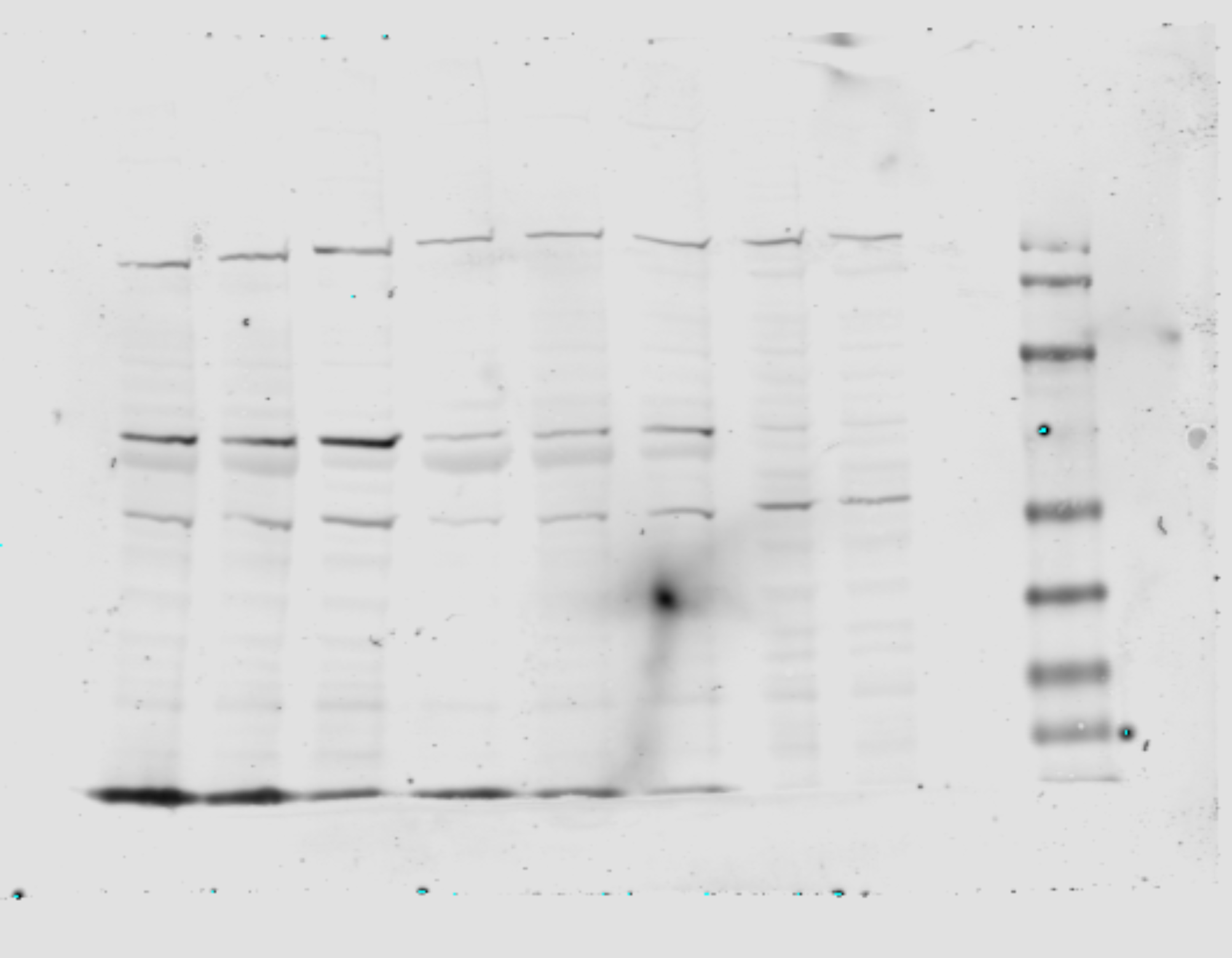

Supplement: Source data 2. [file elife-72171-supp2.zip › SCFA Paper/Supplemental Figure 5B_SourceData1.tif]

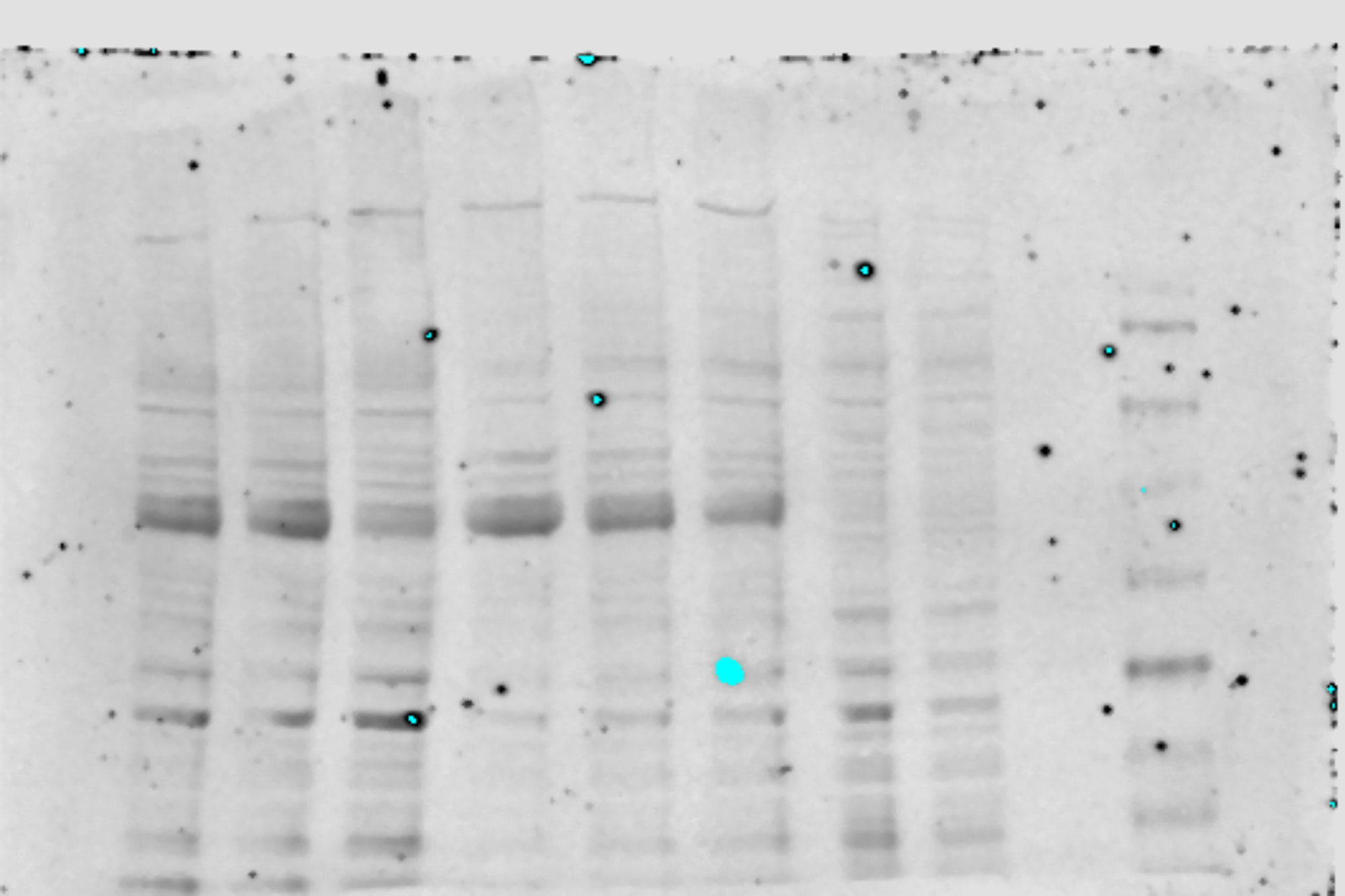

Supplement: Source data 2. [file elife-72171-supp2.zip › SCFA Paper/Supplemental Figure 5B_SourceData2.tif]

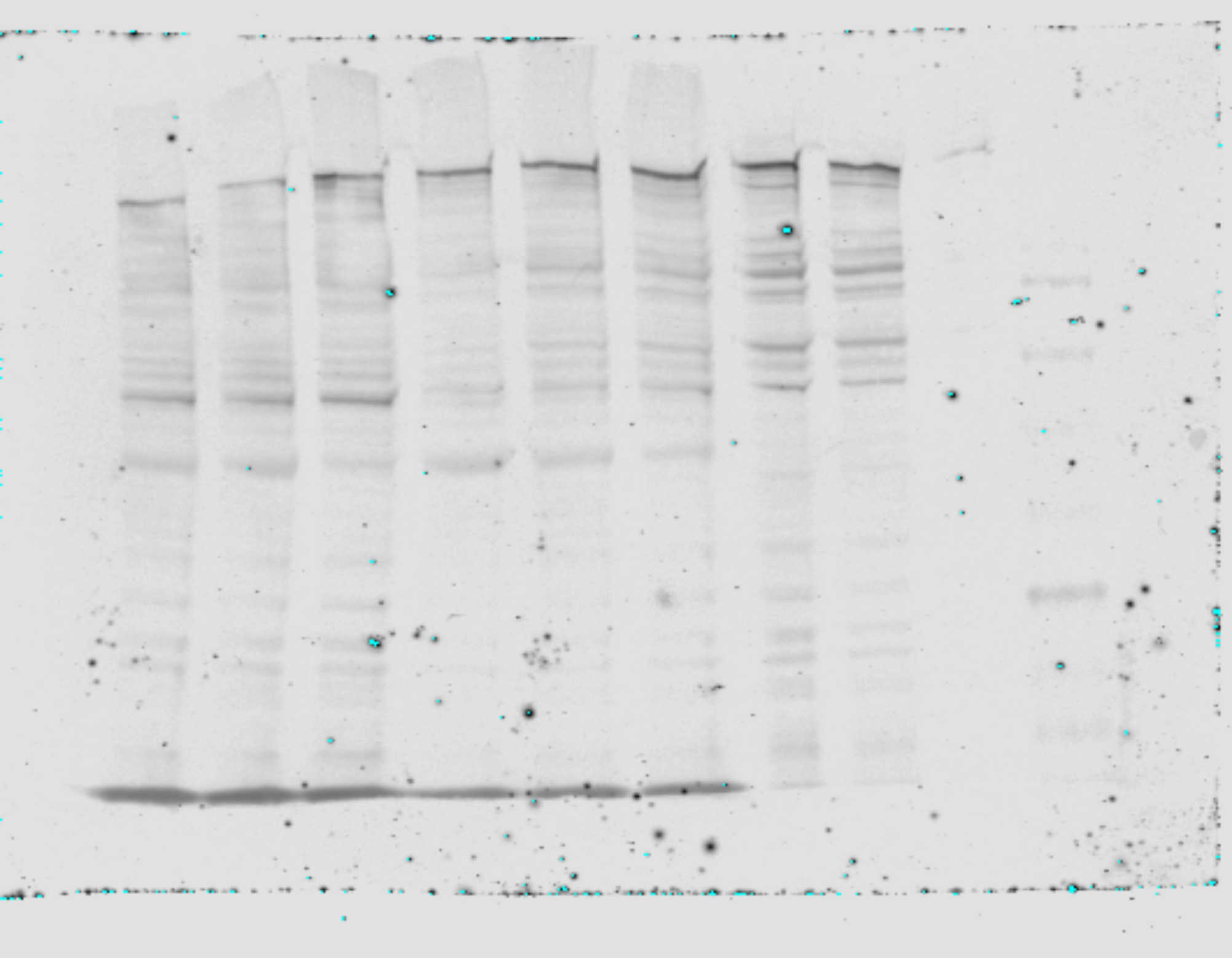

Supplement: Source data 2. [file elife-72171-supp2.zip › SCFA Paper/Supplemental Figure 5B_SourceData3.tif]

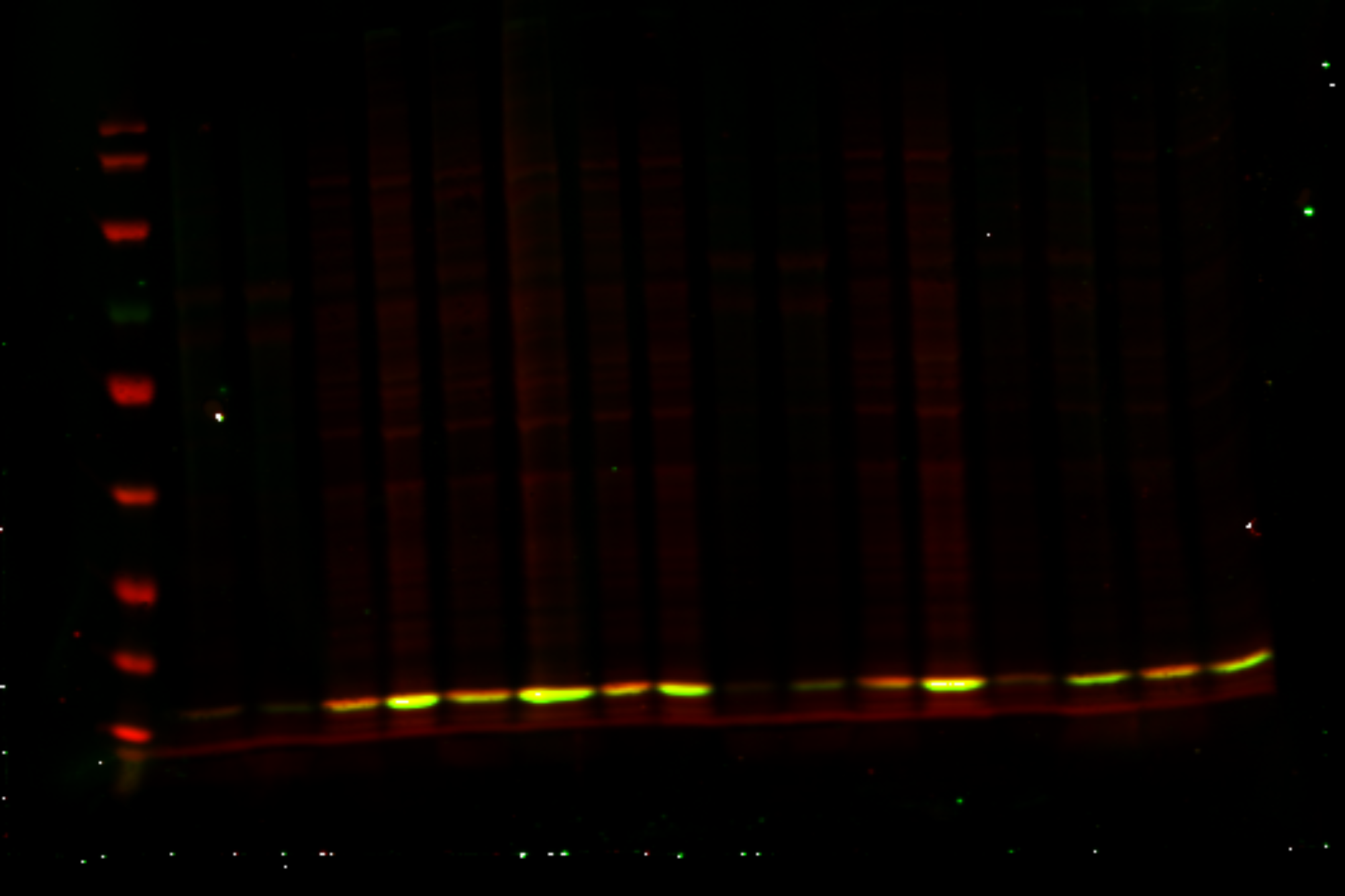

Supplement: Source data 2. [file elife-72171-supp2.zip › SCFA Paper/Supplemental Figure 5C_OtherData1.tif]

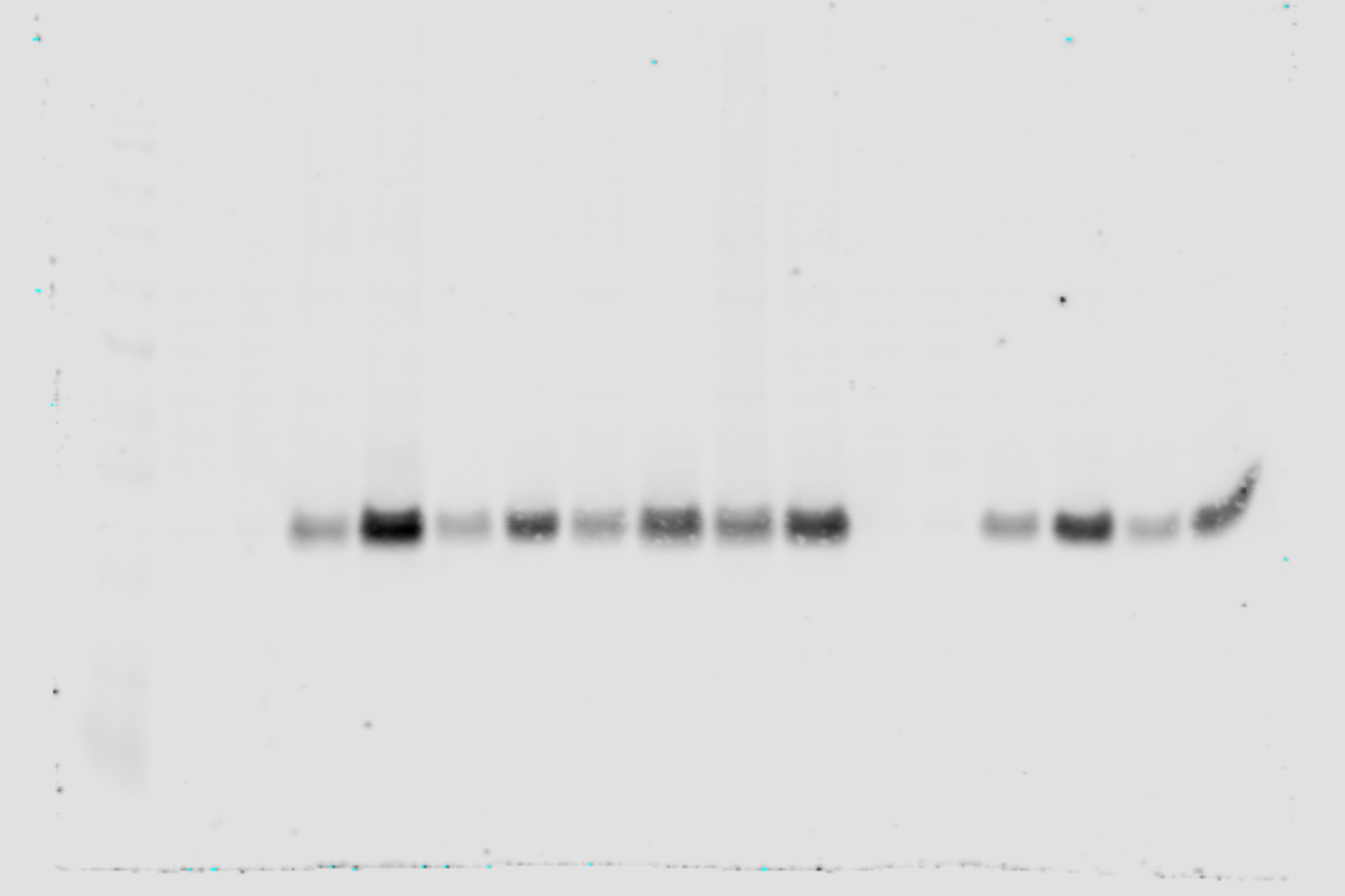

Supplement: Source data 2. [file elife-72171-supp2.zip › SCFA Paper/Supplemental Figure 5C_SourceData1.tif]

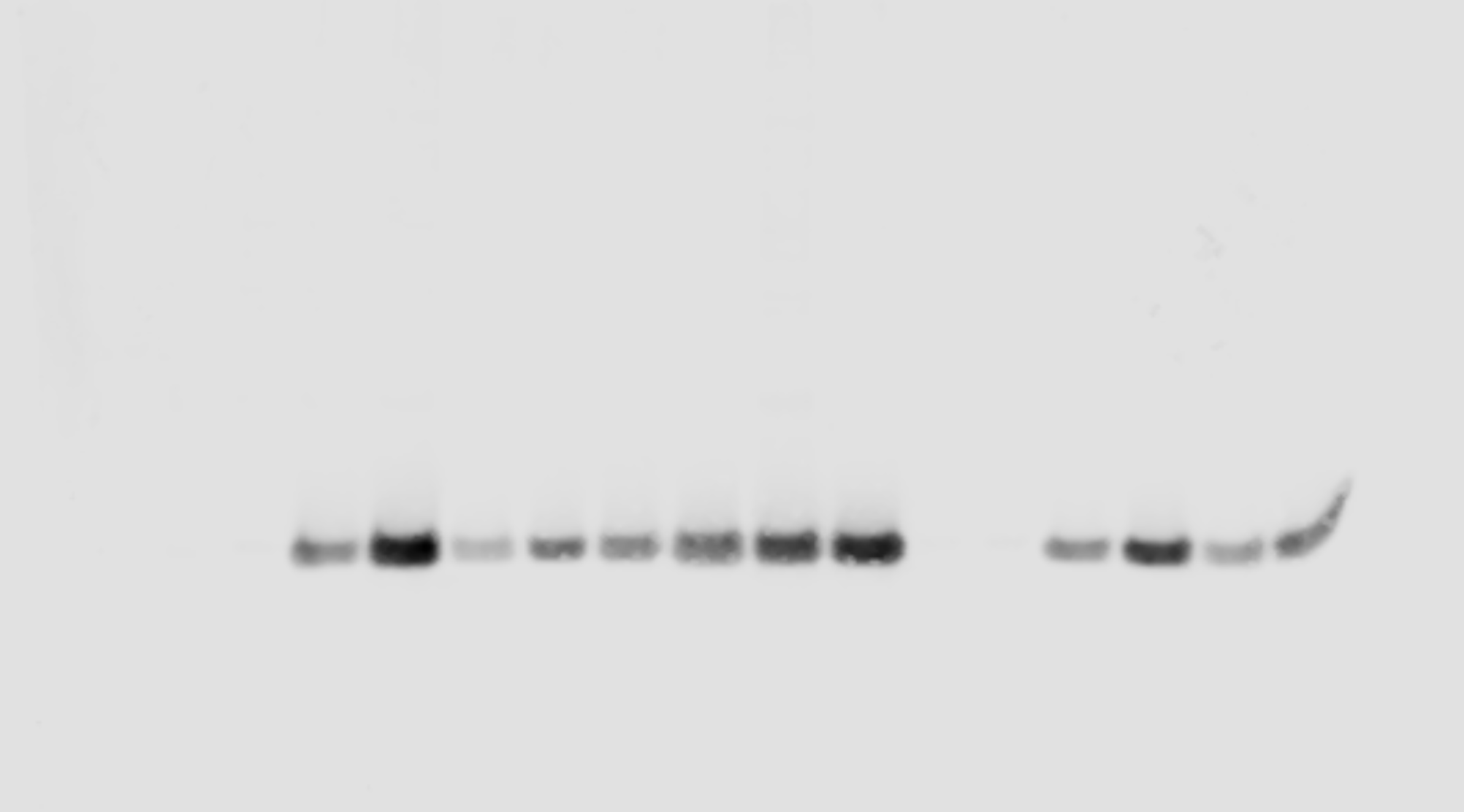

Supplement: Source data 2. [file elife-72171-supp2.zip › SCFA Paper/Supplemental Figure 5C_SourceData2.tif]

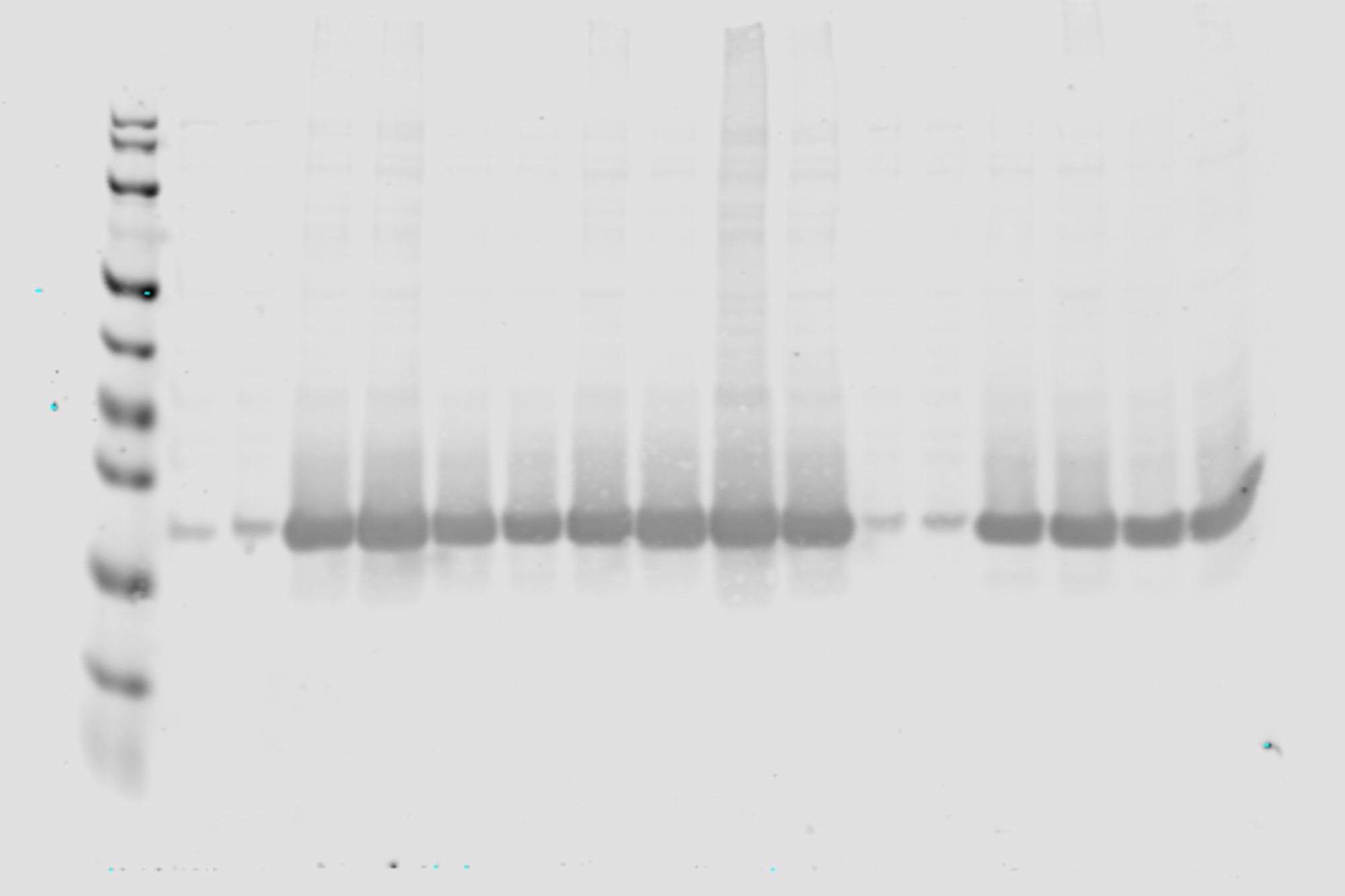

Supplement: Source data 2. [file elife-72171-supp2.zip › SCFA Paper/Supplemental Figure 5C_SourceData3.tif]

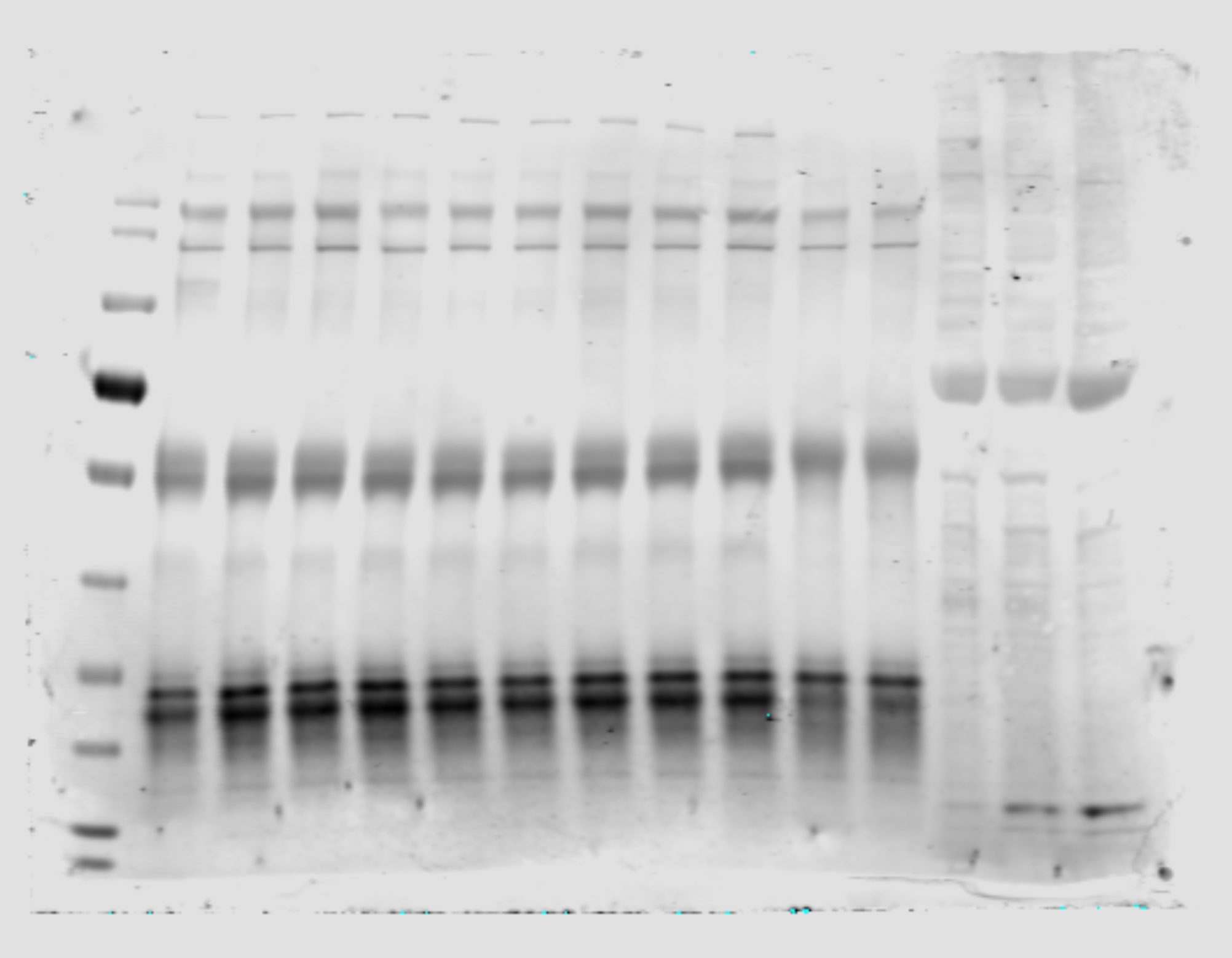

Supplement: Source data 2. [file elife-72171-supp2.zip › SCFA Paper/Supplemental Figure 5D_SourceData1.tif]

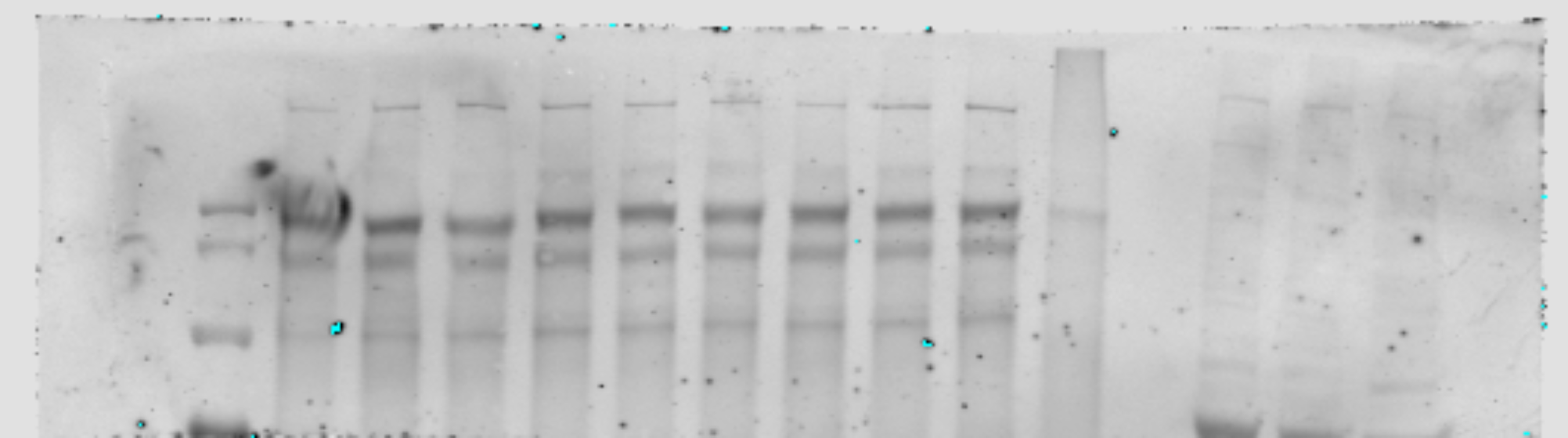

Supplement: Source data 2. [file elife-72171-supp2.zip › SCFA Paper/Supplemental Figure 5D_SourceData2.tif]

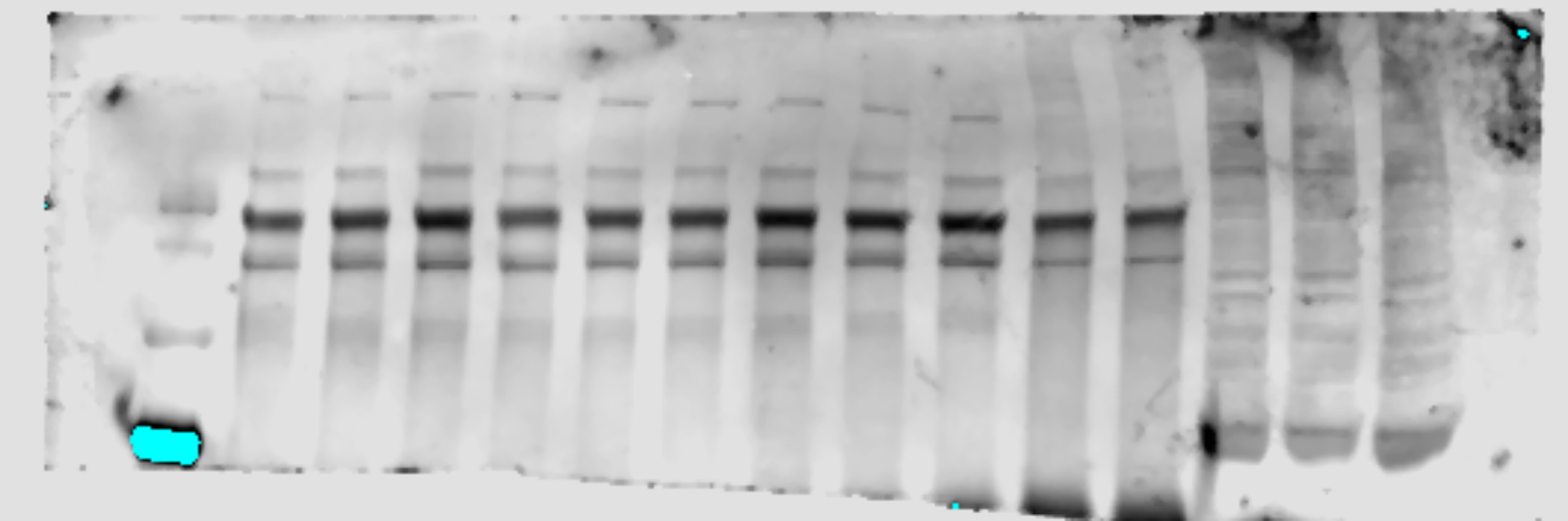

Supplement: Source data 2. [file elife-72171-supp2.zip › SCFA Paper/Supplemental Figure 5D_SourceData3.tif]

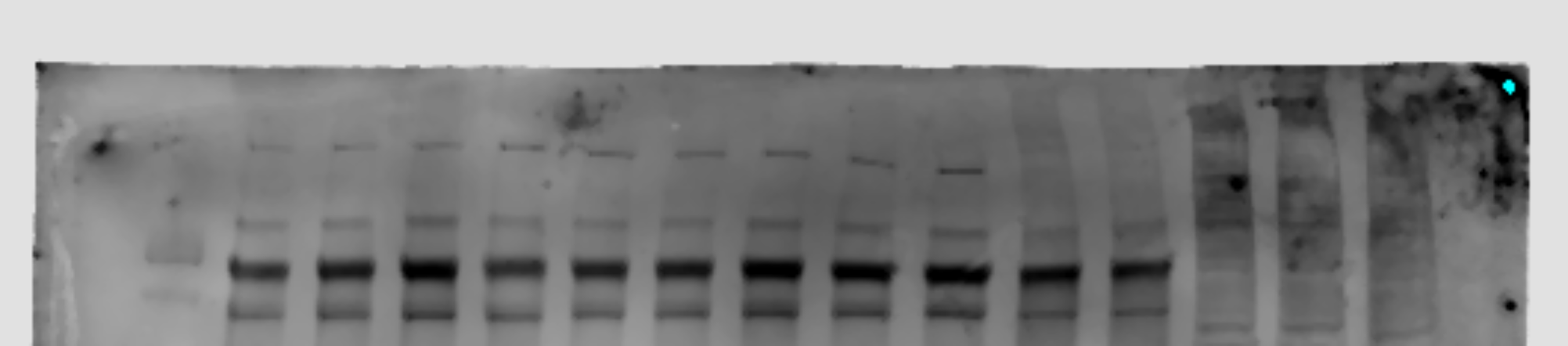

Supplement: Source data 2. [file elife-72171-supp2.zip › SCFA Paper/Supplemental Figure 5D_SourceData4.tif]

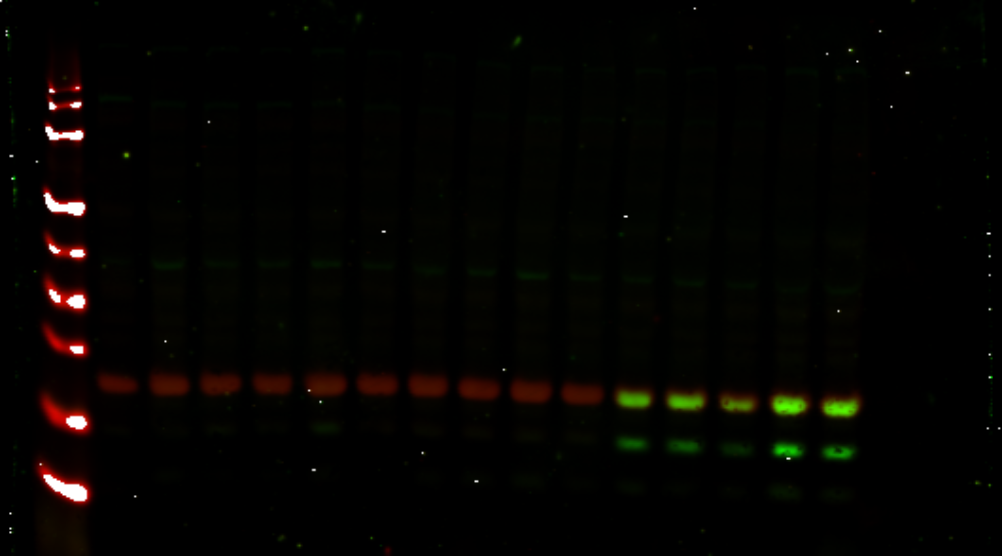

Supplement: Source data 2. [file elife-72171-supp2.zip › SCFA Paper/Supplemental Figure5A_OtherData_1.tif]

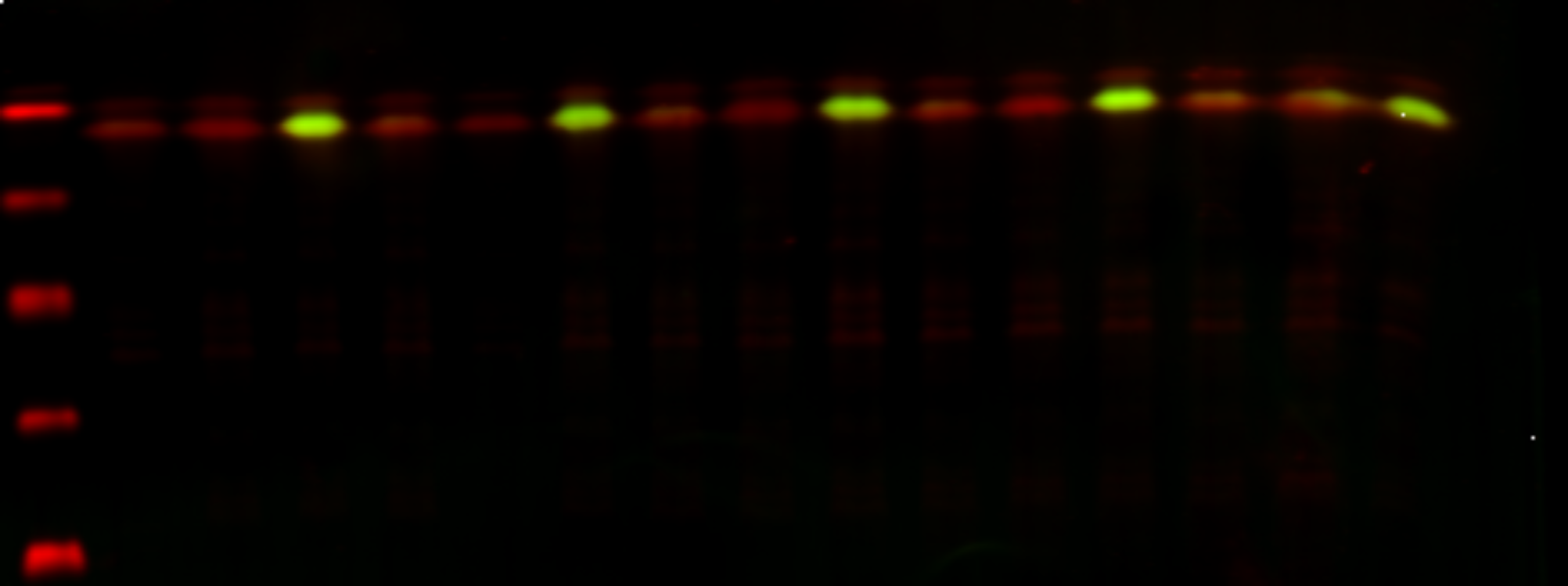

Supplement: Source data 2. [file elife-72171-supp2.zip › SCFA Paper/Supplemental Figure5A_OtherData_10.tif]

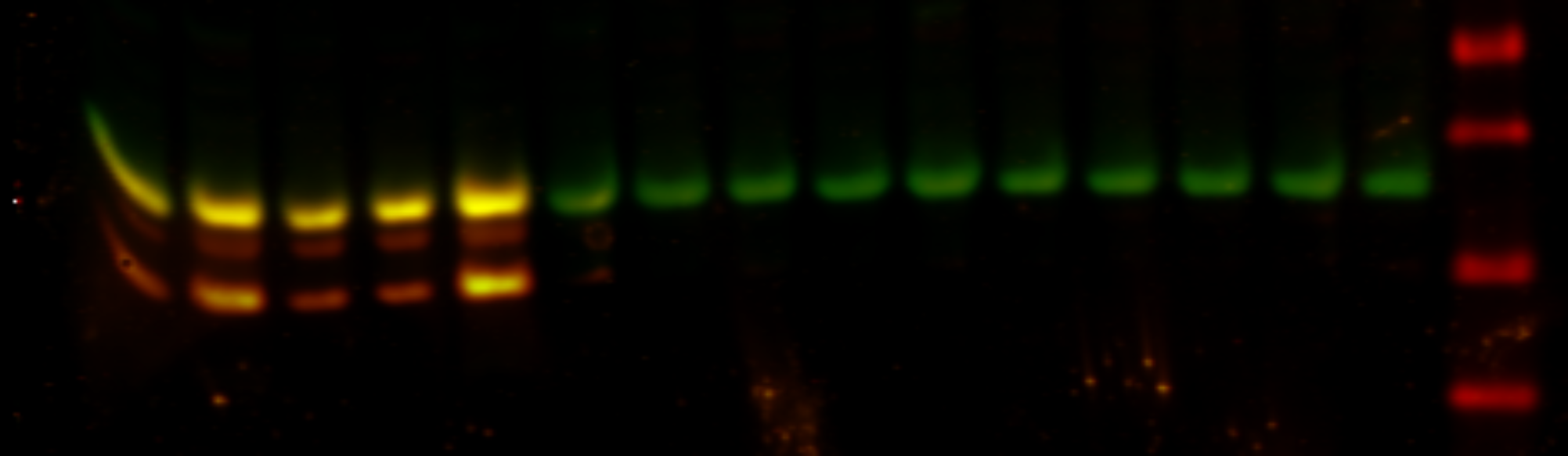

Supplement: Source data 2. [file elife-72171-supp2.zip › SCFA Paper/Supplemental Figure5A_OtherData_11.tif]

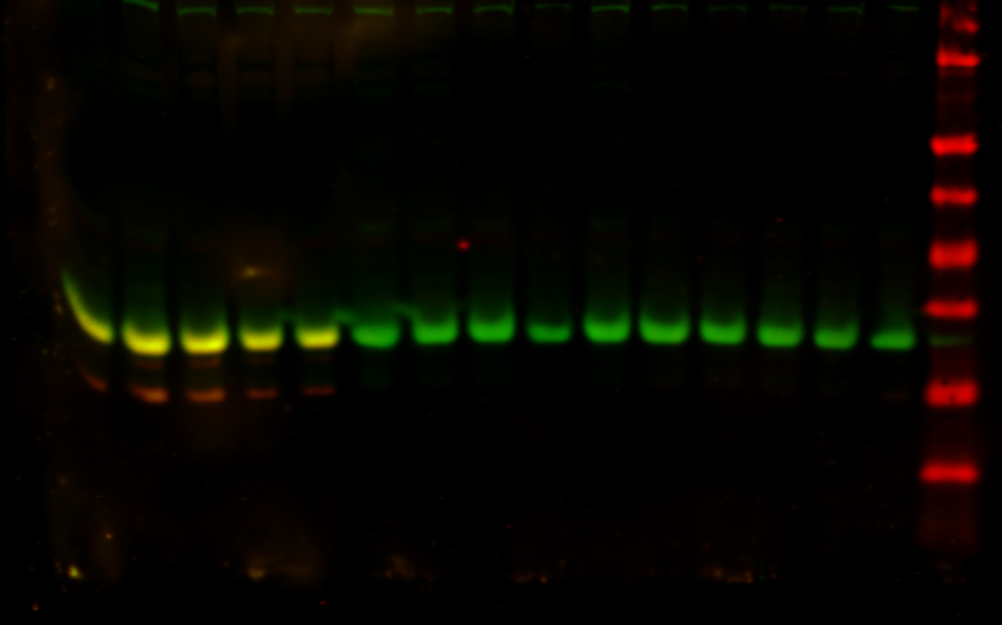

Supplement: Source data 2. [file elife-72171-supp2.zip › SCFA Paper/Supplemental Figure5A_OtherData_12.tif]

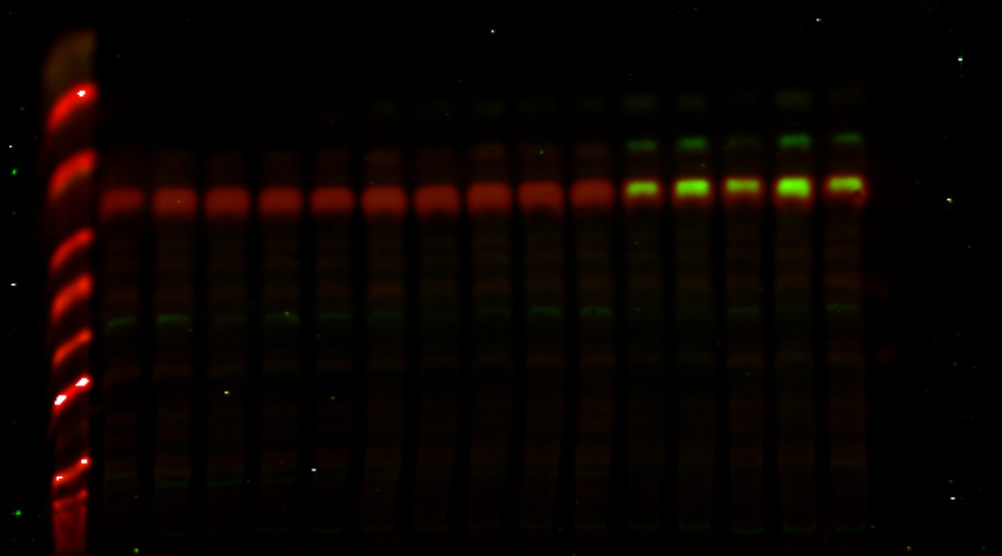

Supplement: Source data 2. [file elife-72171-supp2.zip › SCFA Paper/Supplemental Figure5A_OtherData_2.tif]

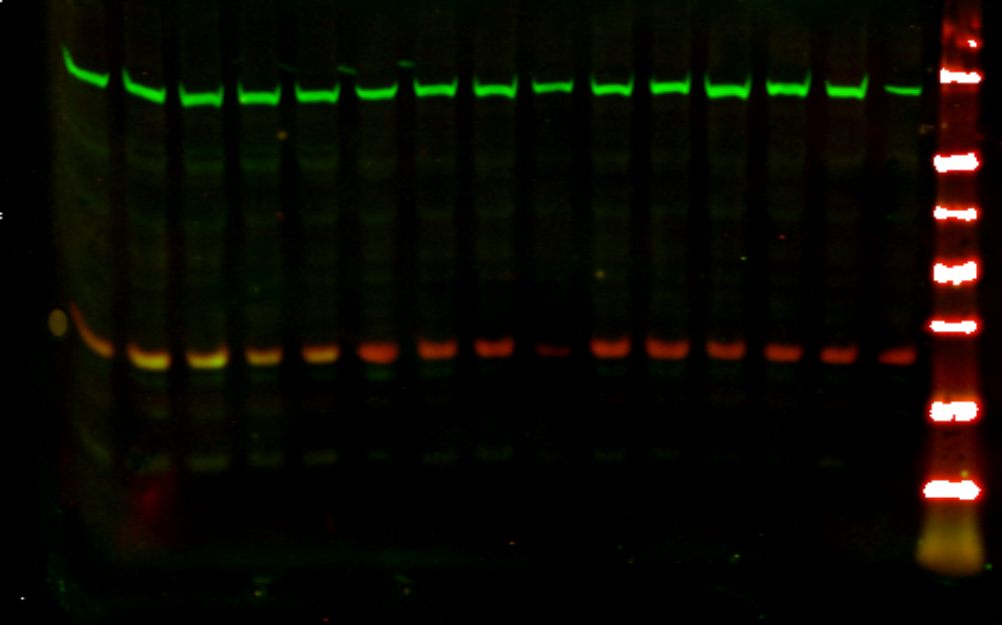

Supplement: Source data 2. [file elife-72171-supp2.zip › SCFA Paper/Supplemental Figure5A_OtherData_3.tif]

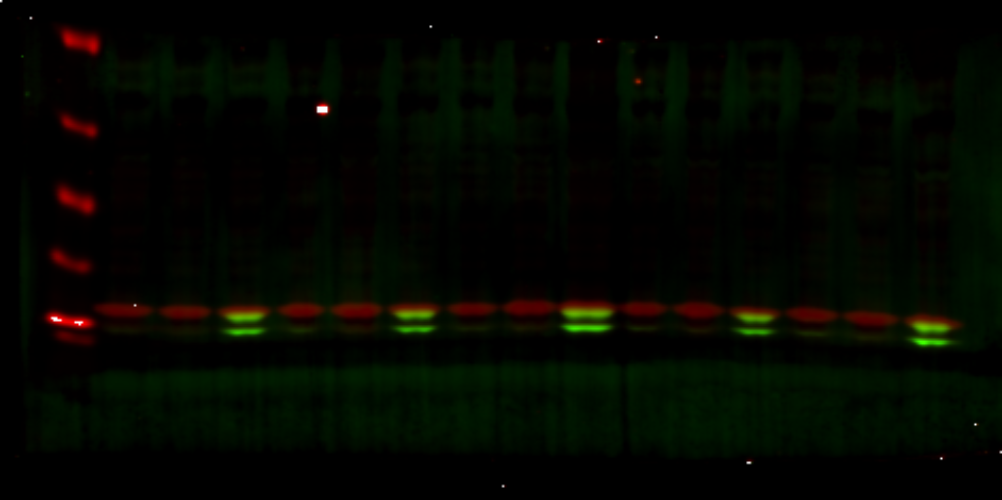

Supplement: Source data 2. [file elife-72171-supp2.zip › SCFA Paper/Supplemental Figure5A_OtherData_5.tif]

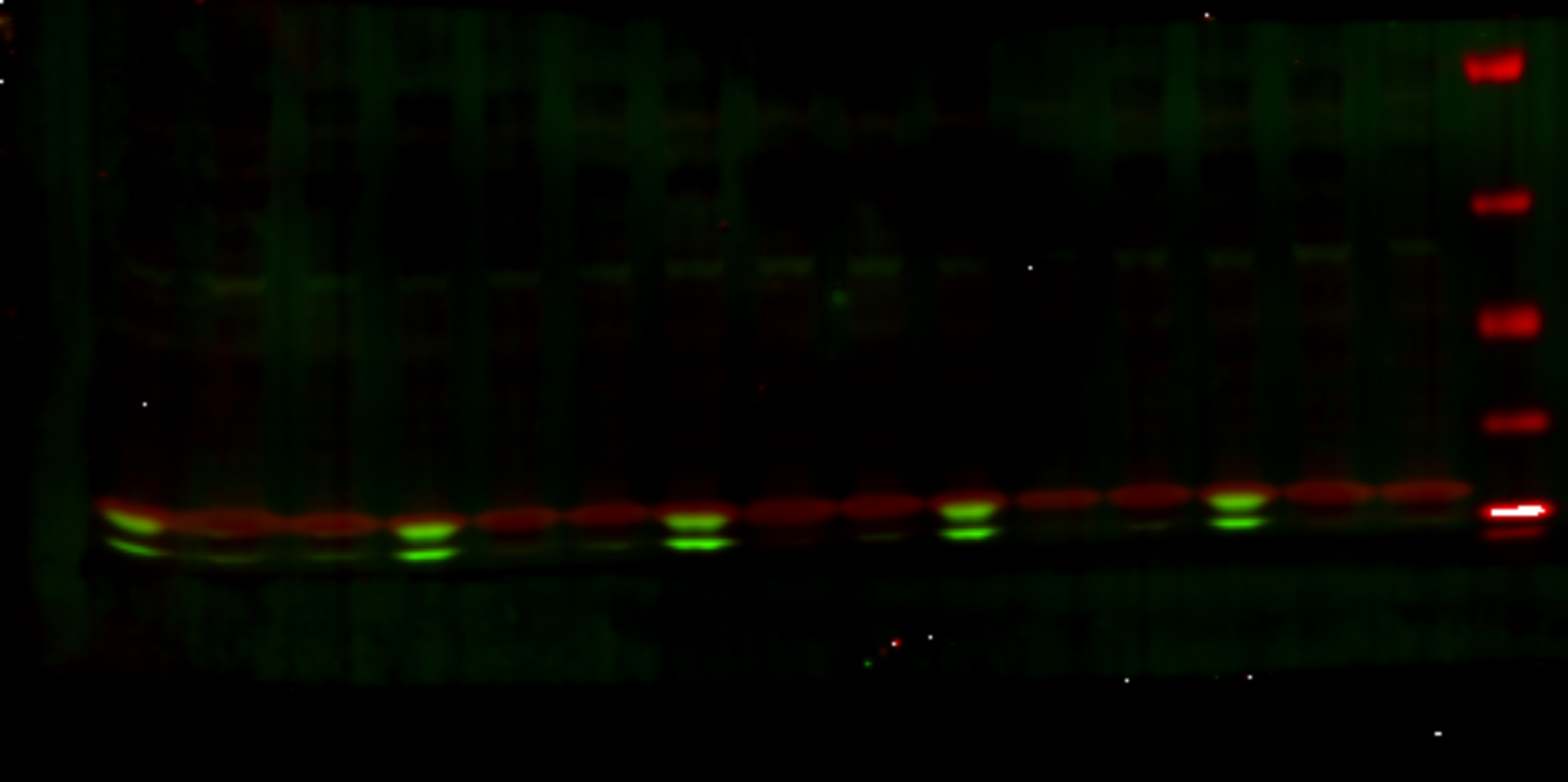

Supplement: Source data 2. [file elife-72171-supp2.zip › SCFA Paper/Supplemental Figure5A_OtherData_6.tif]

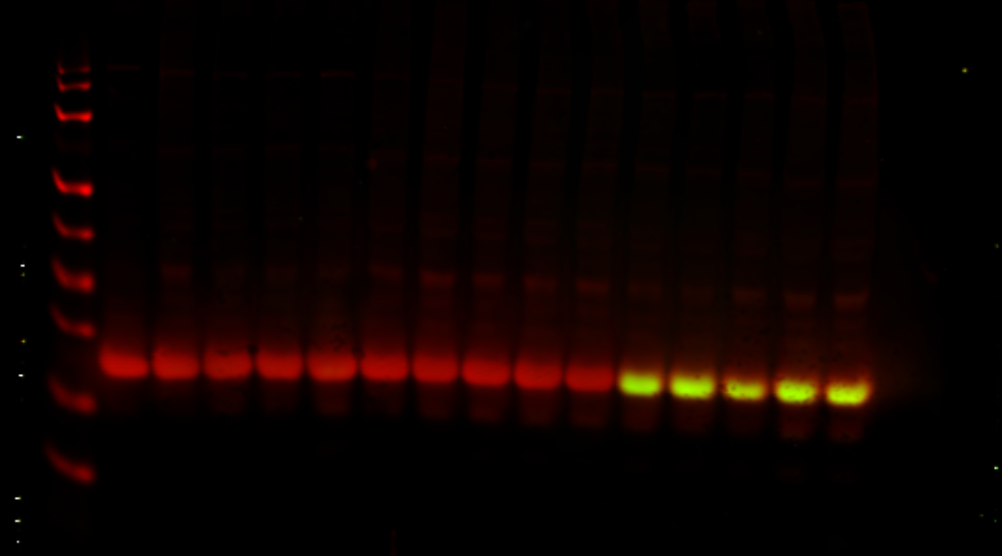

Supplement: Source data 2. [file elife-72171-supp2.zip › SCFA Paper/Supplemental Figure5A_OtherData_7.tif]

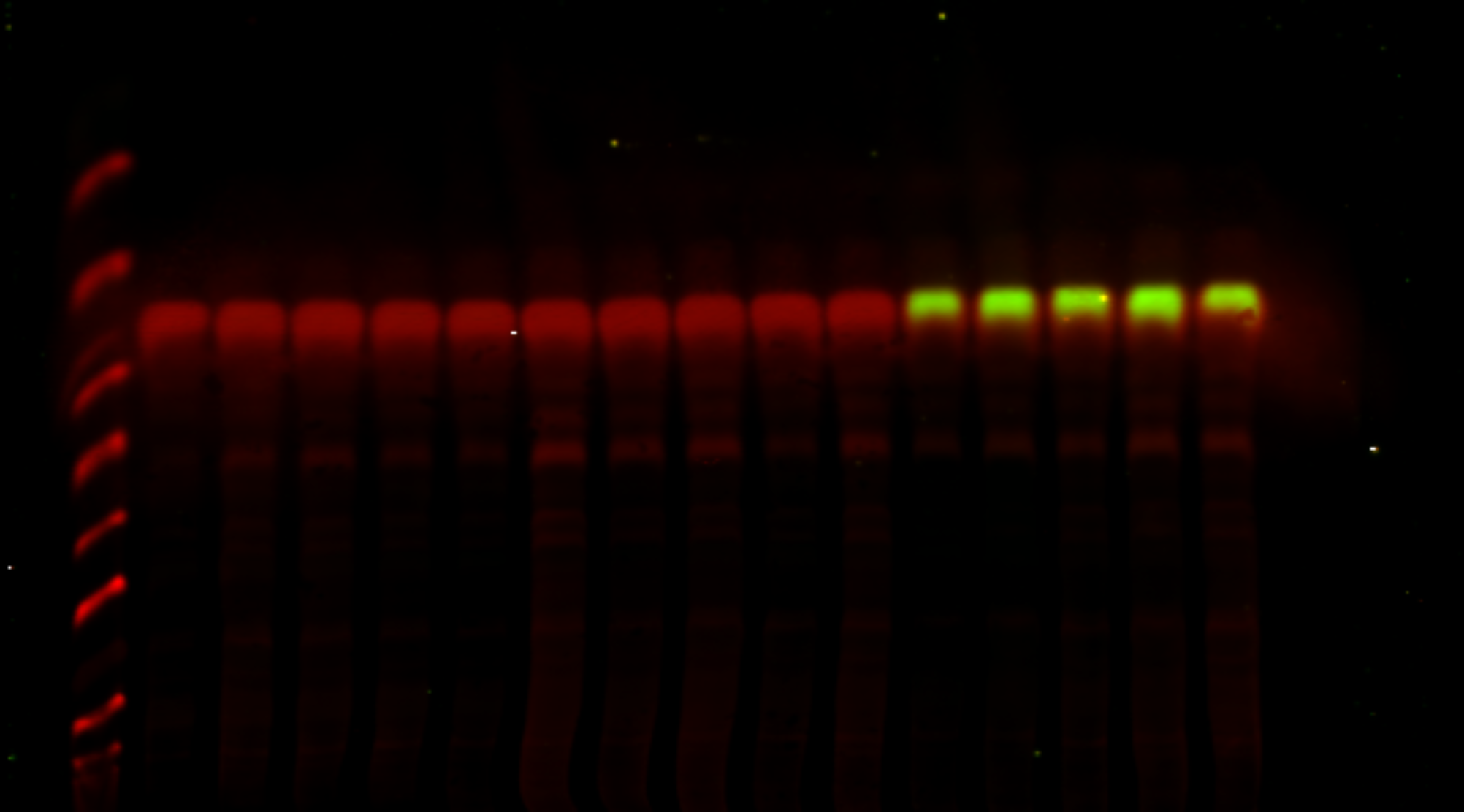

Supplement: Source data 2. [file elife-72171-supp2.zip › SCFA Paper/Supplemental Figure5A_OtherData_8.tif]

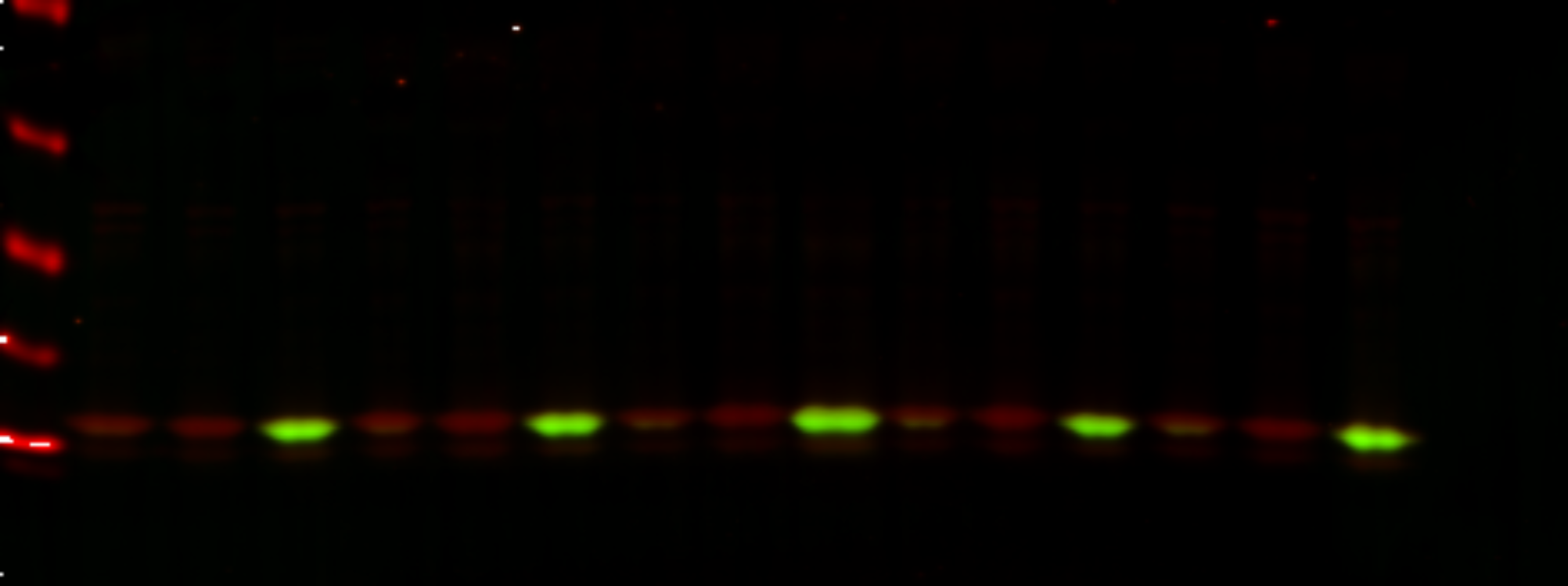

Supplement: Source data 2. [file elife-72171-supp2.zip › SCFA Paper/Supplemental Figure5A_OtherData_9.tif]

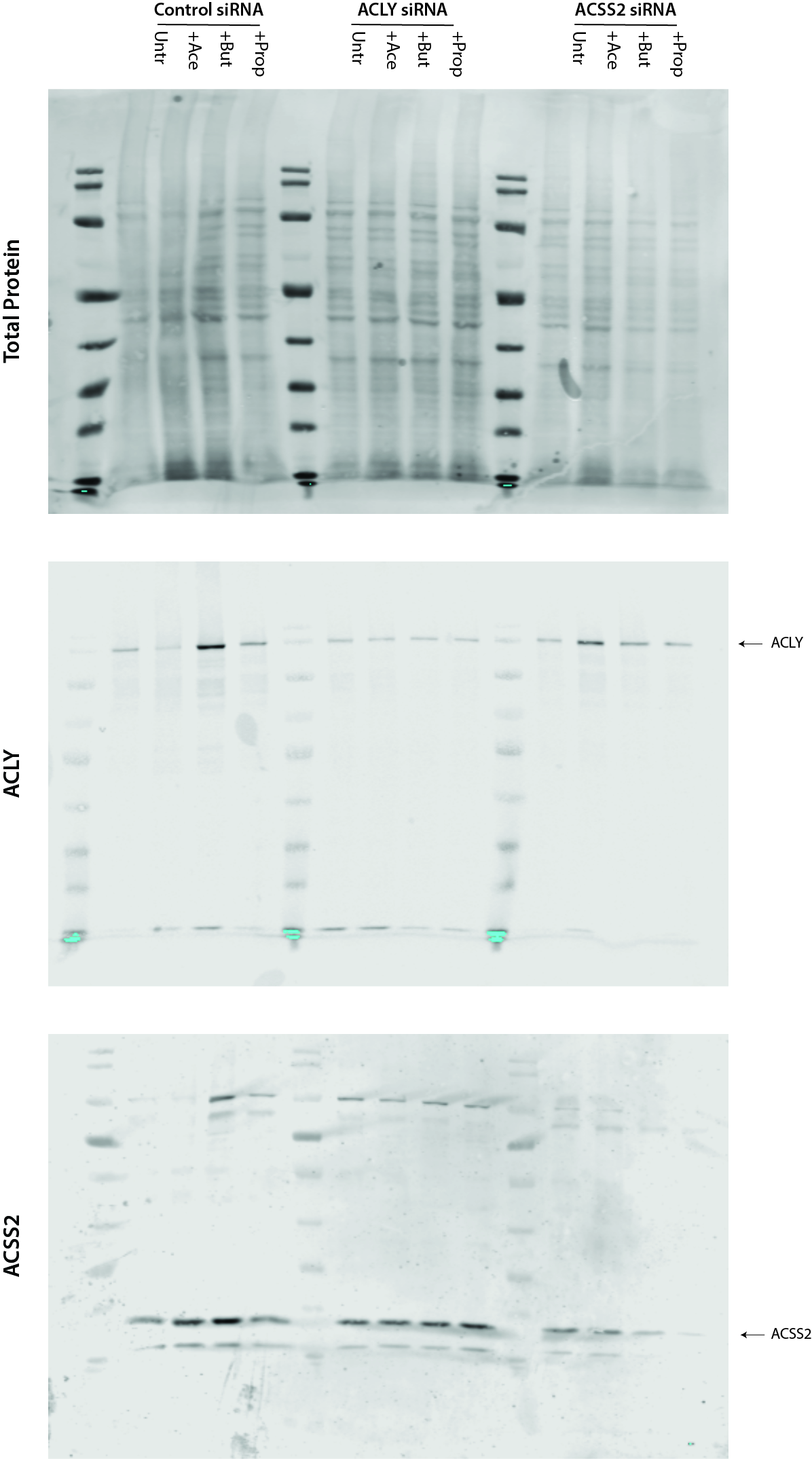

Supplement: Source data 2. [file elife-72171-supp2.zip › SCFA Paper/Supplemental3C_SourceFigure.tif]

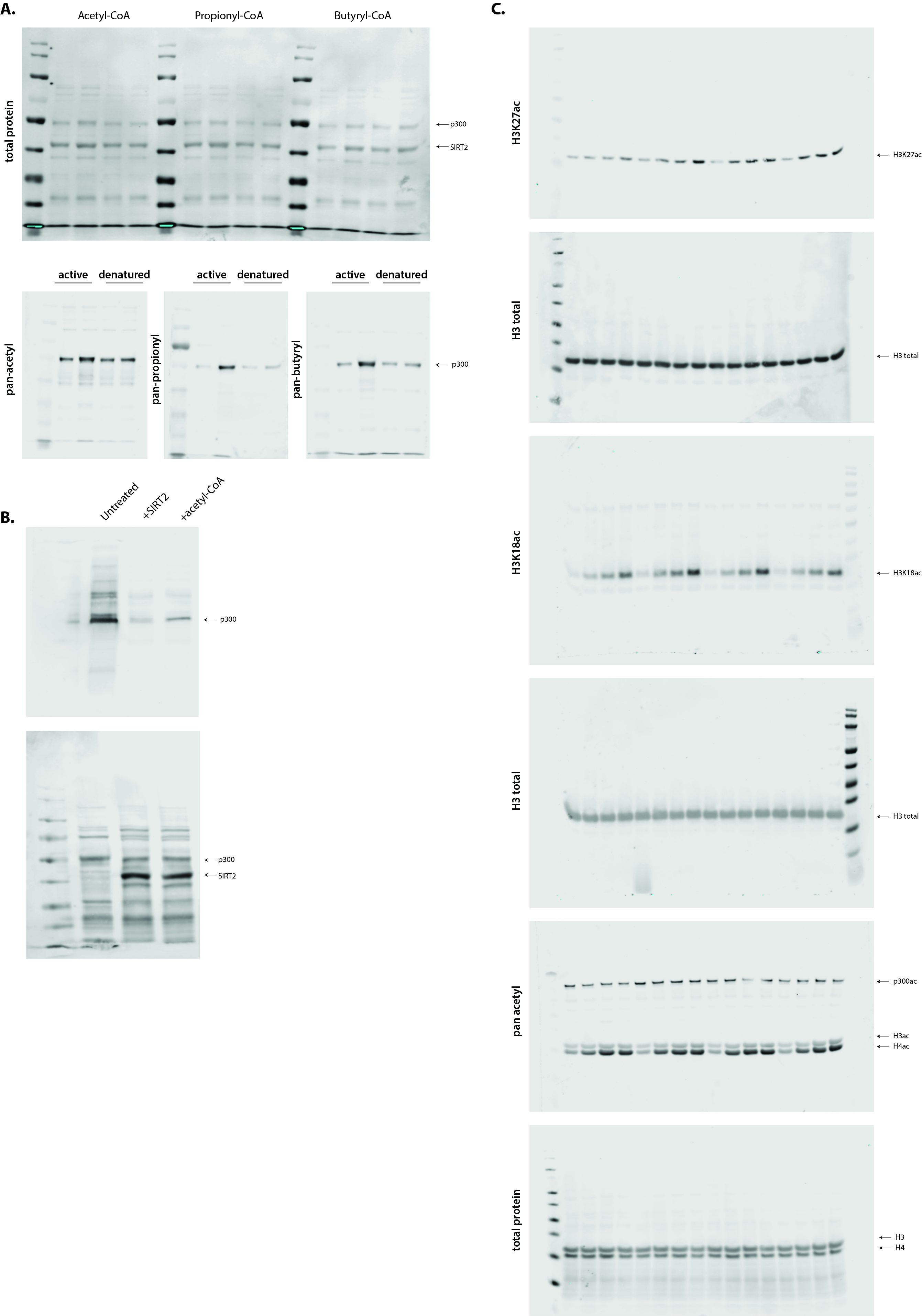

Supplement: Source data 2. [file elife-72171-supp2.zip › SCFA Paper/Supplemental4_SourceFigure.tif]

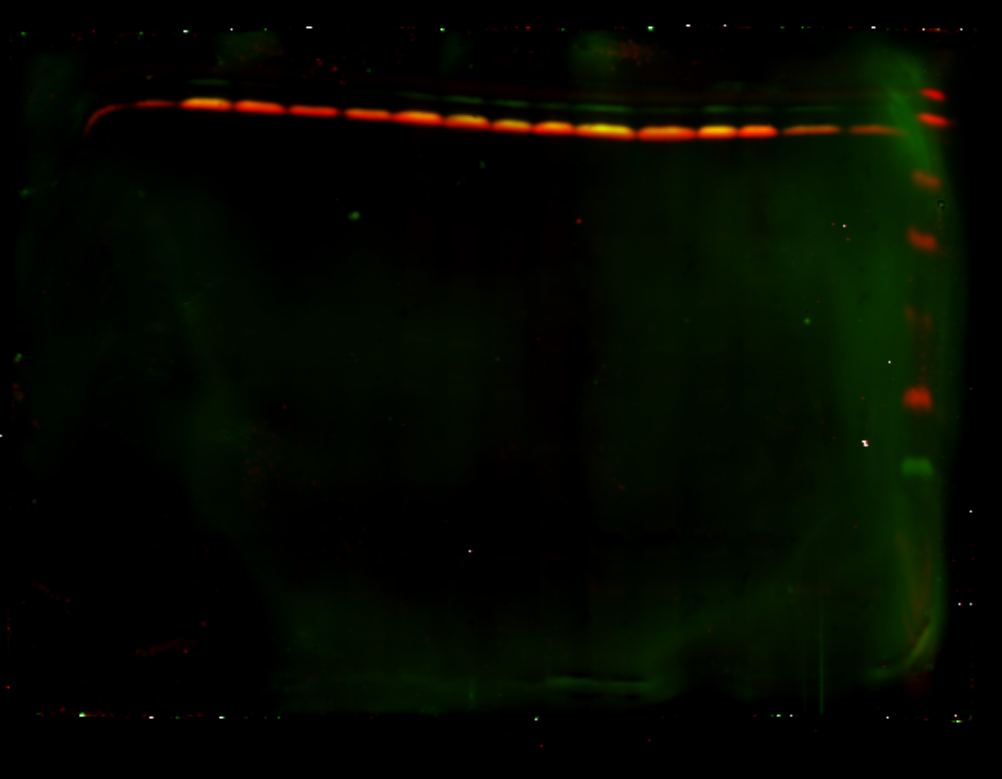

Supplement: Source data 2. [file elife-72171-supp2.zip › SCFA Paper/Supplemetal Figure 5C_OtherData_2.tif]

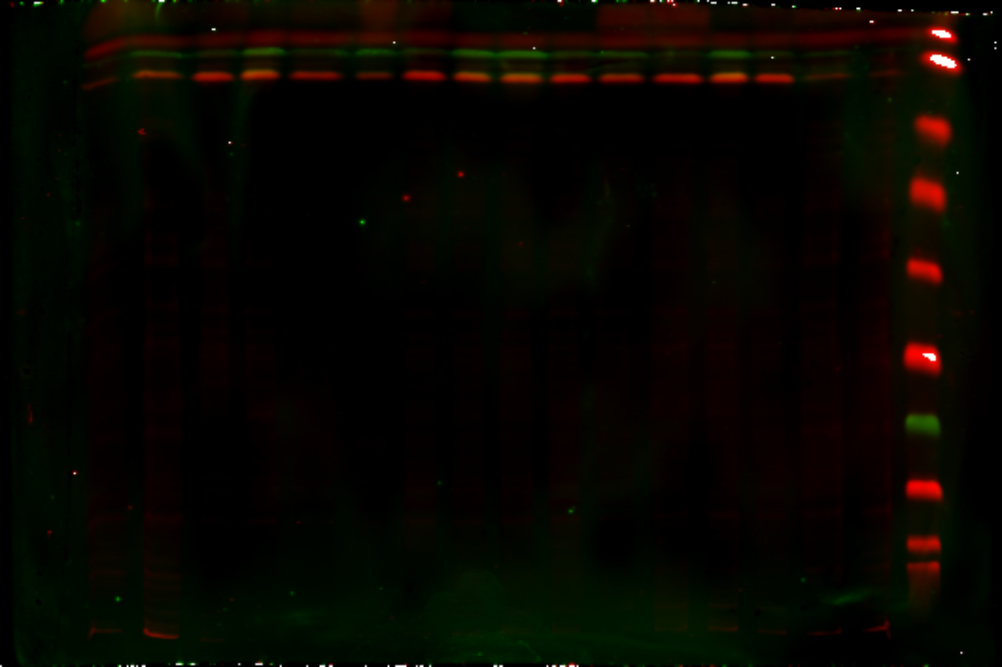

Supplement: Source data 2. [file elife-72171-supp2.zip › SCFA Paper/Supplemetal Figure 5C_OtherData_3.tif]

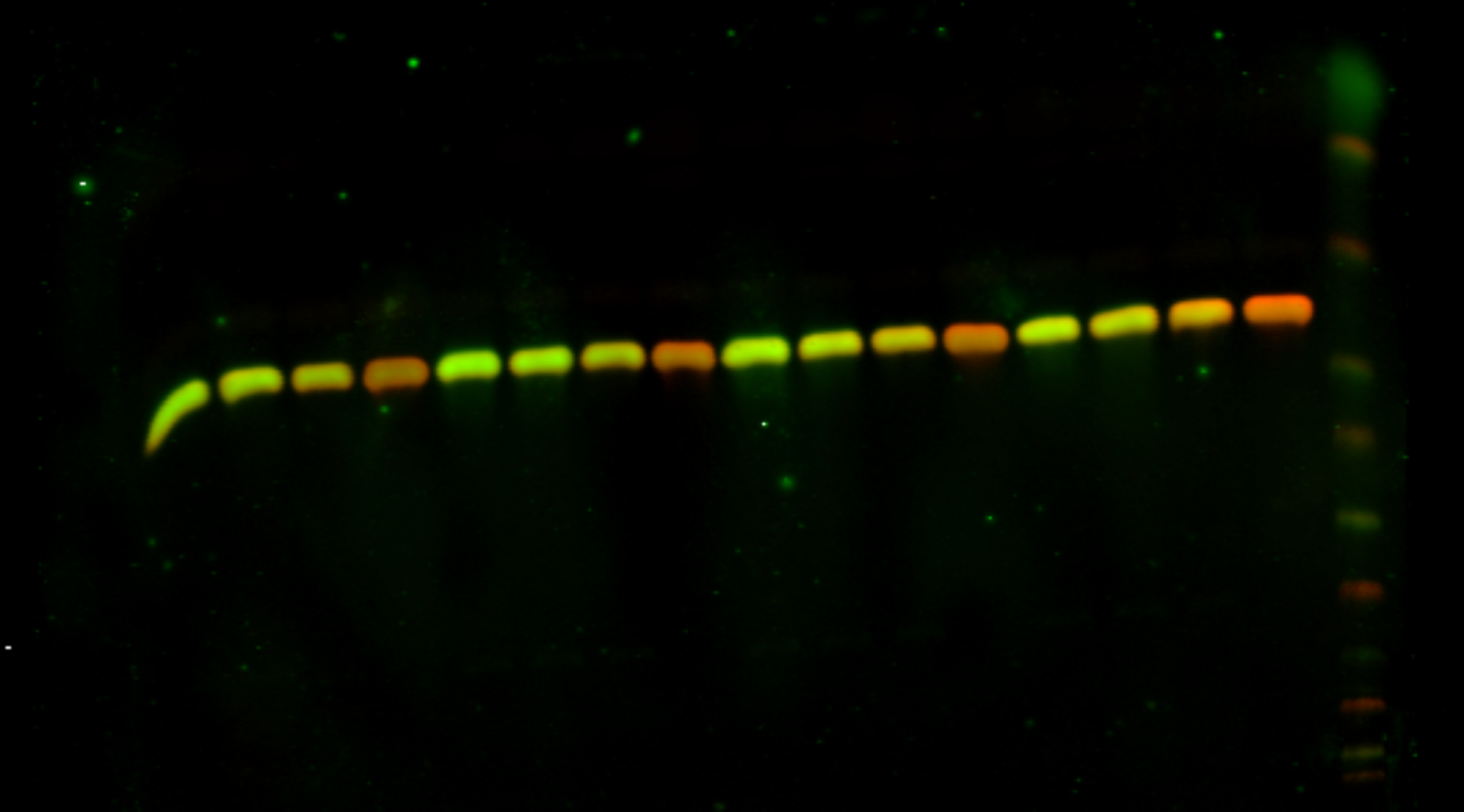

Supplement: Source data 2. [file elife-72171-supp2.zip › SCFA Paper/Supplemetal Figure 5C_OtherData_4.tif]

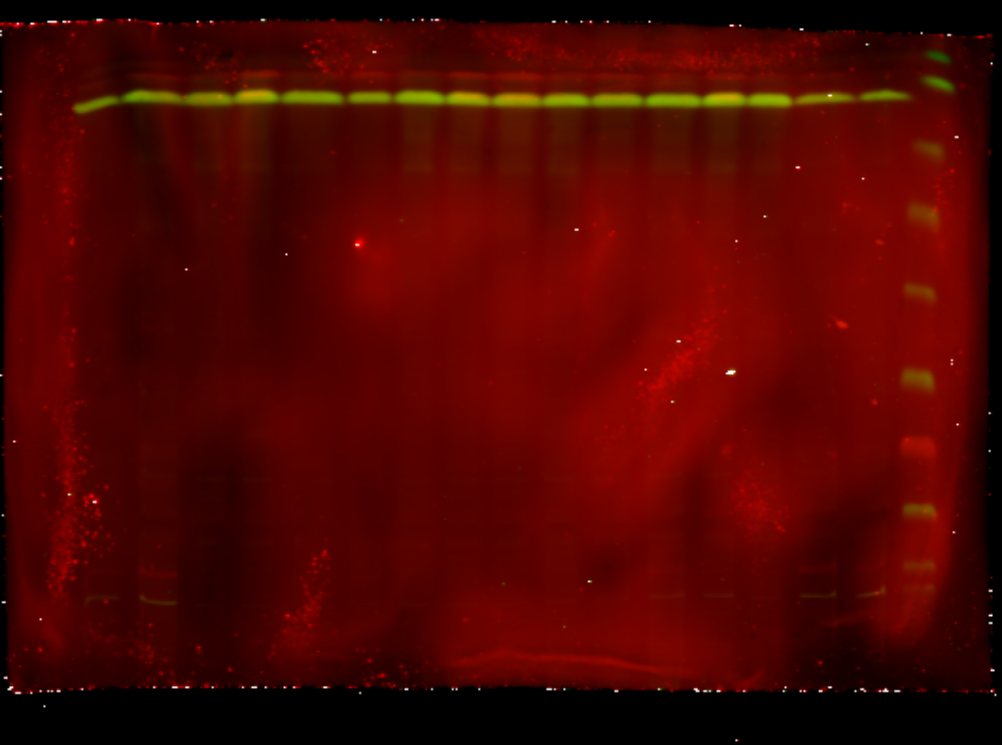

Supplement: Source data 2. [file elife-72171-supp2.zip › SCFA Paper/Supplemetal Figure 5C_OtherData_5.tif]

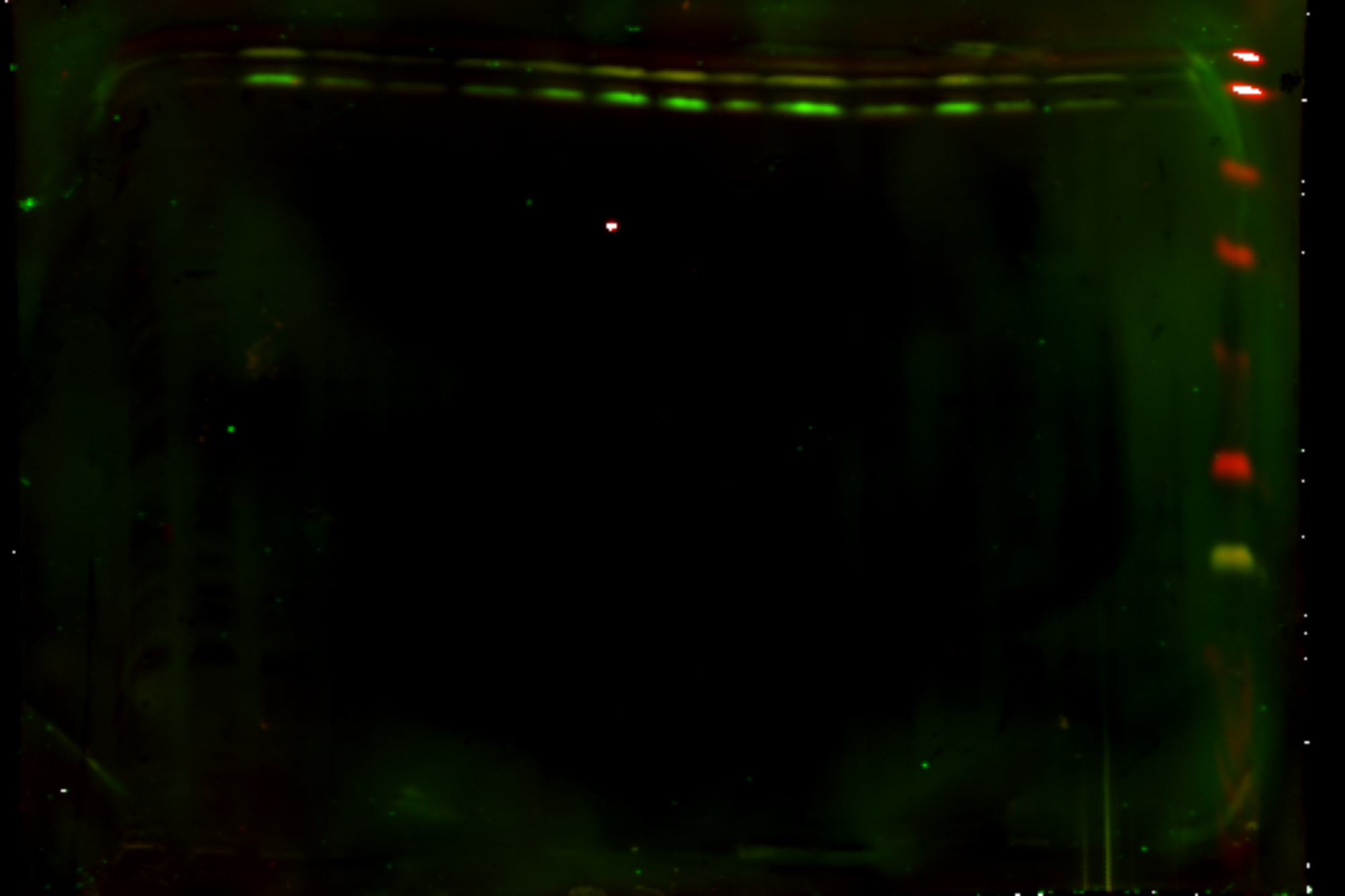

Supplement: Source data 2. [file elife-72171-supp2.zip › SCFA Paper/Supplemetal Figure 5C_OtherData_6.tif]

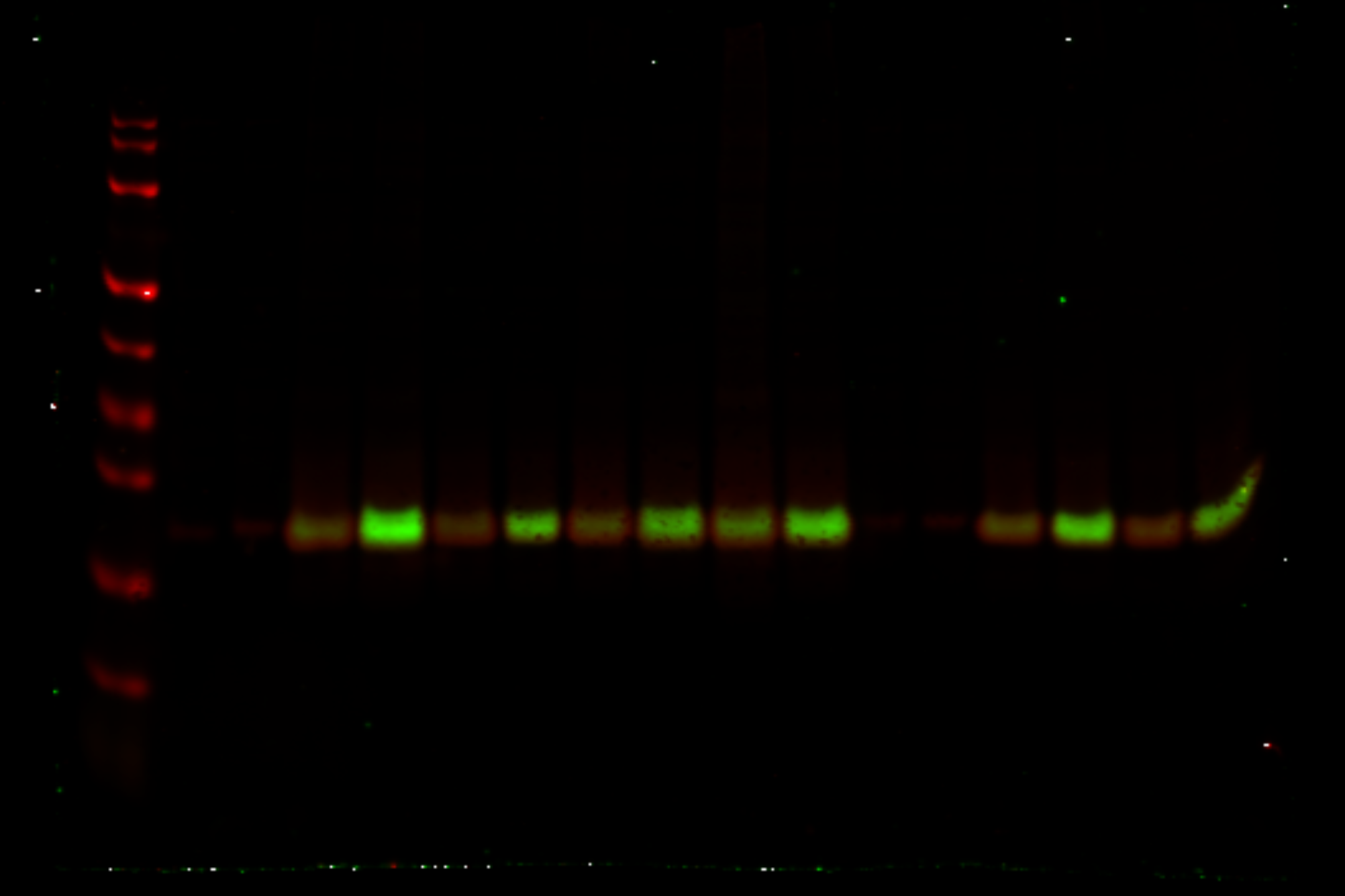

Supplement: Source data 2. [file elife-72171-supp2.zip › SCFA Paper/Supplemetal Figure 5C_OtherData_7.tif]

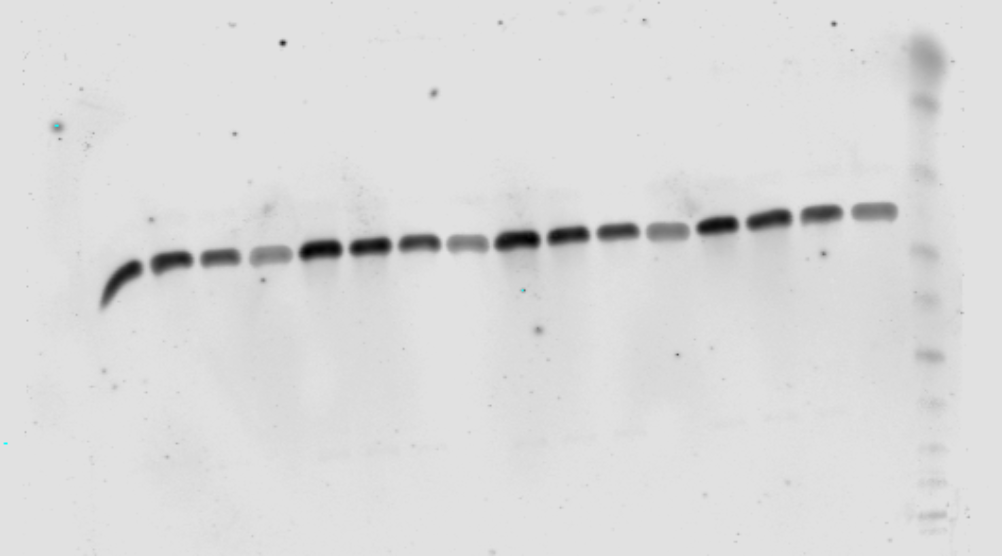

Supplement: Source data 2. [file elife-72171-supp2.zip › SCFA Paper/Supplemetal Figure 5C_OtherData_8.tif]
